# Supplementary material for: Sequence variants associated with BMI affect disease risk through BMI itself
Source: Nat Commun. 2024 Nov 12;15:9335. doi: 10.1038/s41467-024-53568-9 (PMC11557886; doi:10.1038/s41467-024-53568-9)
Supplement: Supplementary file 1 — Supplementary Information [file 41467_2024_53568_MOESM1_ESM.pdf]

Supplementary Information for the Article „Genetically predicted BMI affects disease risk through BMI itself“

Supplementary Notes

Introduction

This supplementary document contains supplementary figures, tables, and brief notes on specific details in the analysis that did not fit in the main manuscript. This includes information regarding nonlinear effects and derivation of theoretical effects stemming from adjustments for a measured exposure in the GRS outcome association analysis from a gaussian directed acyclic graph. We furthermore include mediation analysis of the outliers exclusively and we describe in more detail the smoking adjustment.

Nonlinear relationship between BMI and diseases

Since we observed differences in the effect estimates of the genetic scores and the BMI phenotype, specifically for heart failure and myocardial infarction in UK Biobank, we investigated the BMI phenotype associations further. The relationship between BMI and HF risk suggests that for BMI in the range 20-23 kg/m^2, the risk is near constant (Supplementary Figure 22). This pattern is not consistent with the log-linear link between BMI and disease risk which is the functional relationship assumed by our models. We decided to reassess the associations after excluding low and high BMI. We tried excluding individuals with BMI below 25 kg/m^2 at first visit and those with BMI over 35 kg/m^2 at first visit (Supplementary Figure 23 and Supplementary Table 9). The HF association is clearly sensitive to the exclusion of individuals with BMI lower than 25 kg/m^2, as the OR for the entire dataset is 1.11 but 1.15 when individuals with BMI under 25 kg/m^2 are excluded.

Mediation due to partial observation of exposure and spurious associations of BMI-GRS with outcomes through confounders

In order to understand what can influence the GRS effect, e.g. confounders, we derive theoretical regression coefficients under simplistic models using the assumptions from Mendelian Randomization.

Mathematical interpretation of the GRS association after adjustment for a measured exposure

The causal diagram in Supplementary Figure 28a) represents a directed acyclic graph (DAG) for a structural equation model (SEM) that we define as follows:

1) $g \sim \mathcal{N}(0,1)$
2) $C \sim \mathcal{N}(0,1)$
3) $N \sim \mathcal{N}(0,\sigma^2)$
4) $E = \beta_0 \cdot g + \beta_1 \cdot C + N$
5) $Y = \beta_3 \cdot E + \beta_2 \cdot C$

Here g represents a genetic instrument, in our case a genetic risk score, E represents the exposure, Y represents the outcome, C represents confounders that can affect the exposure and the outcome, and N represents residual variation in the exposure, which does not directly affect the outcome.

Notably, here we assume that there are no arrows directly from g to the confounders or the outcome, these assumptions are the 2nd and 3rd Mendelian Randomization (MR) assumptions. Assuming that this model is correct, we can test for a causal effect of the exposure on the outcome by regressing Y on g. The genetic risk score is independent of the confounders, and since the confounders and the genetic risk score collide on the exposure, there is no bias from confounders in the estimate of the causal effect.

We are interested in assessing how much of the genetic association is mediated through the measured exposure. In this case, since we observe E perfectly, we would expect full mediation when we condition on E. However, when we condition on E, we open the backdoor path (g -> E -> C -> Y).

By conditioning on E we also block the path directly from the exposure to the outcome, so if we regress Y on g and E jointly, we should observe an effect of g on Y. This is a form of collider bias since the variables g, E and C form a fork. Given the SEM we can compute the coefficients of the regression directly as:

$$\mathbb{E}[Y|\boldsymbol{E},\boldsymbol{g}] = \left(\beta_3 + \left(\frac{\beta_2 \cdot \beta_1}{\beta_1^2 + \sigma^2}\right)\right) \cdot \boldsymbol{E} - \left(\frac{\beta_2 \cdot \beta_1}{\beta_1^2 + \sigma^2}\right) \cdot \beta_0 \cdot \boldsymbol{g}$$

Thus, the magnitude of the $\boldsymbol{g}$ coefficient in the equation above scales in a complex manner with all the β parameters, except β_3, and σ^2. Since the formula for the coefficient of $\boldsymbol{g}$ does not include β_3, we can say that the association is spurious and the association of $\boldsymbol{g}$ is perfectly mediated through $\boldsymbol{E}$.

Incomplete observation of the exposure

The exposure variable in the SEM is a theoretical construct, and it is very unlikely that we would ever observe such a variable perfectly. The graph in Supplementary Figure 28b) depicts a more realistic scenario, where we include the variable E_{obs} which is the observed exposure with noise; we can also think of it as an incomplete observation of E. An SEM for the graph in 1b) is the same as for the graph in 1a), except we add the following:

6) $E_{obs} = E + N_0$

where N_0 is normally distributed with mean zero and variance σ_0^2. We can now perform the same exercise, where we regress Y on g and E_{obs} jointly. E_{obs} is a child of the fork, so we still open up the backdoor path through the colliders by conditioning on E_{obs}, however, now we are no longer completely blocking the path directly from the exposure to the outcome. This yields the following equation for the regression coefficients:

$$\mathbb{E}[Y|\boldsymbol{E_{obs}},\boldsymbol{g}] =$$

$$\left(\beta_3 \cdot \left(1 - \frac{\sigma_0^2}{\beta_1^2 + \sigma^2 + \sigma_0^2}\right) + \left(\frac{\beta_2\beta_1}{\beta_1^2 + \sigma^2 + \sigma_0^2}\right)\right) \cdot \boldsymbol{E_{obs}} + \left(\frac{\beta_3\beta_0\sigma_0^2 - \beta_2\beta_1\beta_0}{\beta_1^2 + \sigma^2 + \sigma_0^2}\right) \cdot \boldsymbol{g}$$

This equation is more general than the one for the first SEM and the first regression equation is a special case, as can be seen by setting σ_0^2 to zero. The other limit, i.e., when σ_0^2 tends to infinity is also interesting. In that case, the coefficient for $\boldsymbol{g}$ tends to $\beta_3\beta_0$, which is the unadjusted effect, in which case we would interpret the results as none of the asociation is mediated through the measured exposure. We can also see that the added noise parameter further attenuates the effect of the collider bias, since σ_0^2 appears in the denominator.

Interpretation for BMI and diseases

In the case of BMI as an exposure and the outcomes that we investigate, it is likely that β_1 and β_2 have the same sign. In other words, if the confounders increase the BMI, they likely also increase the risk of the diseases. Thus, the total sign of the collider bias introduced by conditioning on BMI is negative. This is important, since it allows us to interpret the magnitude of the attenuation in the mediation analysis as an upper bound on disease risk, and that this bound is most likely a conservative upper bound.

The part of the association that can be attributed to the backdoor path is also scaled by the variance term $\sigma^2 + \sigma_0^2$, which further attenuates the potential bias via confounding.

Note that when we condition on the confounders, we completely block the backdoor path. Practically, it is impossible to do this, both because we do not know all the confounders, but also because like BMI, we do not observe the confounders perfectly. If we condition on incomplete observation of the confounders, we can never completely close the backdoor path. We can assess the impact of adding potential confounders to the regression, to investigate the sensitivity of the parameter estimates in the regressions of the genetic risk score on the diseases with BMI as a covariate.

Adding additional variables can have other impacts on the results than just diminishing the effect that goes through the backdoor path. These variables could also include information that are not captured by the measured BMI.

Mediation Analysis Using Exclusively Outliers

Since many of the outlier variants likely represent reverse causation, BMI measurements act as a collider for those variants. To exemplify this phenomenon, we have constructed a GRS exclusively from the outlier variants and redone the mediation analysis (Supplementary Figure 24). Note that the ORs are for 1 SD increase in BMI. Knee replacement is the only outcome that convincingly shows results somewhat consistent with the GRS in the main mediation analysis, (hip replacement and heart failure are somewhat consistent with large CIs), since BMI is likely acting on knee replacement, through sheer mechanical force on the joint, it is likely that the underlying mechanism that creates diversity in BMI is less important in that case.

Most of the other diseases do not associate with this outlier GRS and when we condition on BMI, the effect tends away from the null, this is likely a spurious association that stems from conditioning on a collider. This is most drastic in the case of T2D where we see an association with an effect ($\log(OR) < 0$) of opposite sign to the GRS and the adjusted effect is even further away from the null.

Adjustment for smoking

To adjust for smoking we included three smoking-related terms in the regression models. Smoking is a complex behavioral phenotype, and we wanted to adjust for heavy smoking. The three terms were:

- Smoking status, which is a factor variable that can take the values current, previous and never.
- Pack years, which was transformed to take the value pack years minus twenty if pack years was less than 20, zero otherwise.
- Years since stopped smoking, this variable was transformed in the same way as pack years.

Supplementary Figures

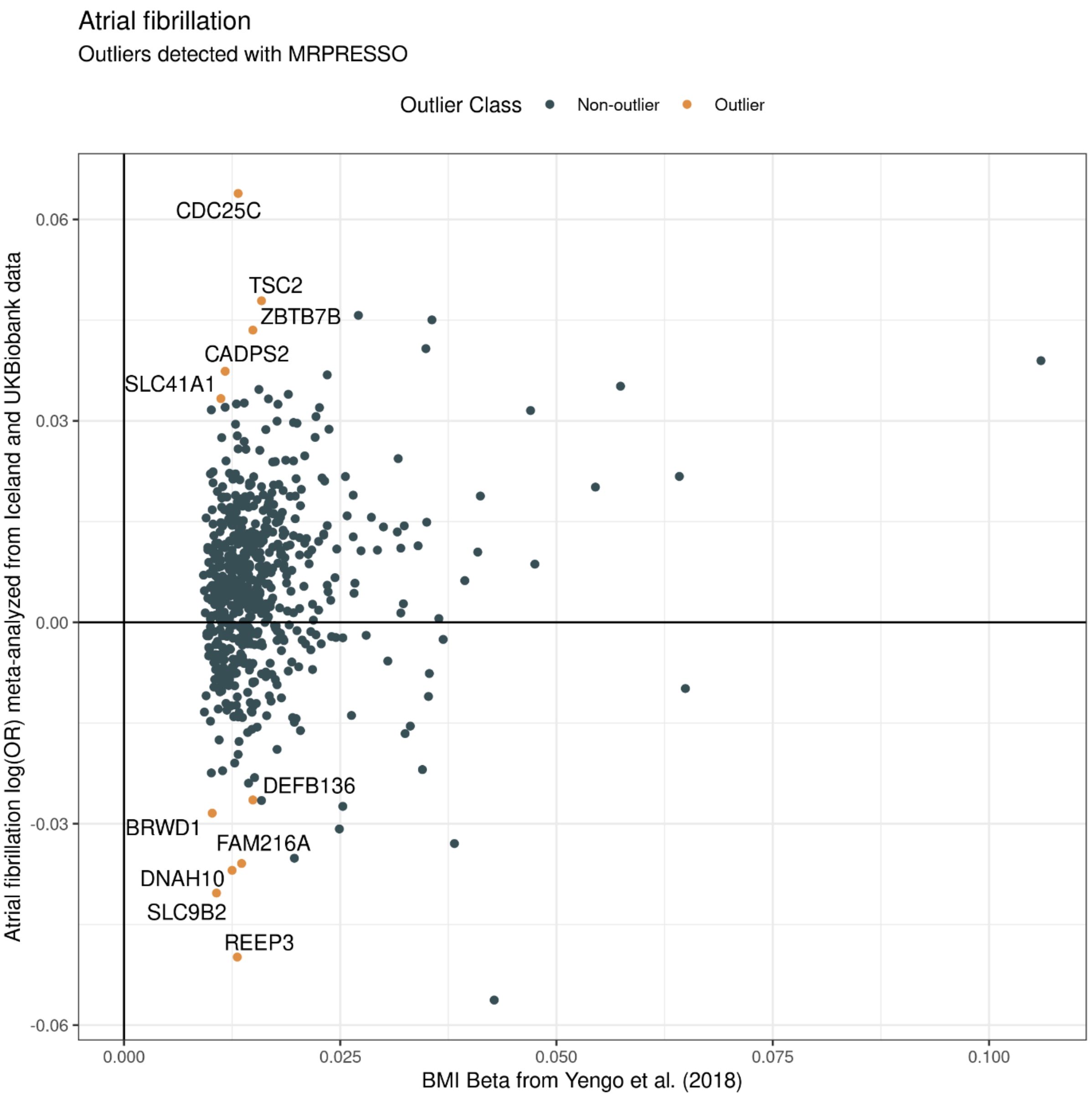

Supplementary Figure 1 Scatter plot showing effects of BMI variants on BMI on the x-axis and their effects (log(OR)) on atrial fibrillation on the y-axis. Outliers detected with MRPRESSO are colored in orange and annotated with the closest gene.

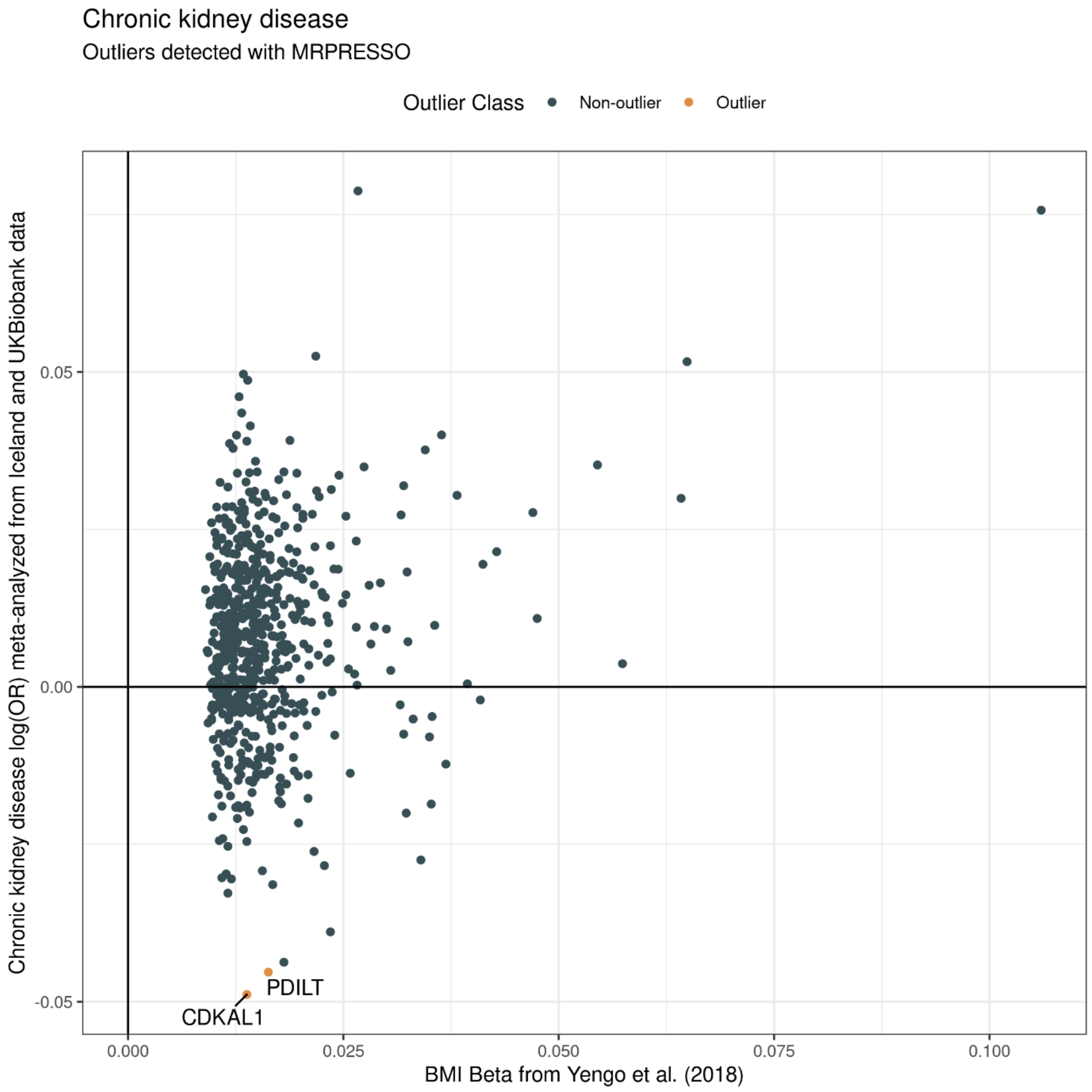

Supplementary Figure 2 Scatter plot showing effects of BMI variants on BMI on the x-axis and their effects (log(OR)) on chronic kidney disease on the y-axis. Outliers detected with MRPRESSO are colored in orange and annotated with the closest gene.

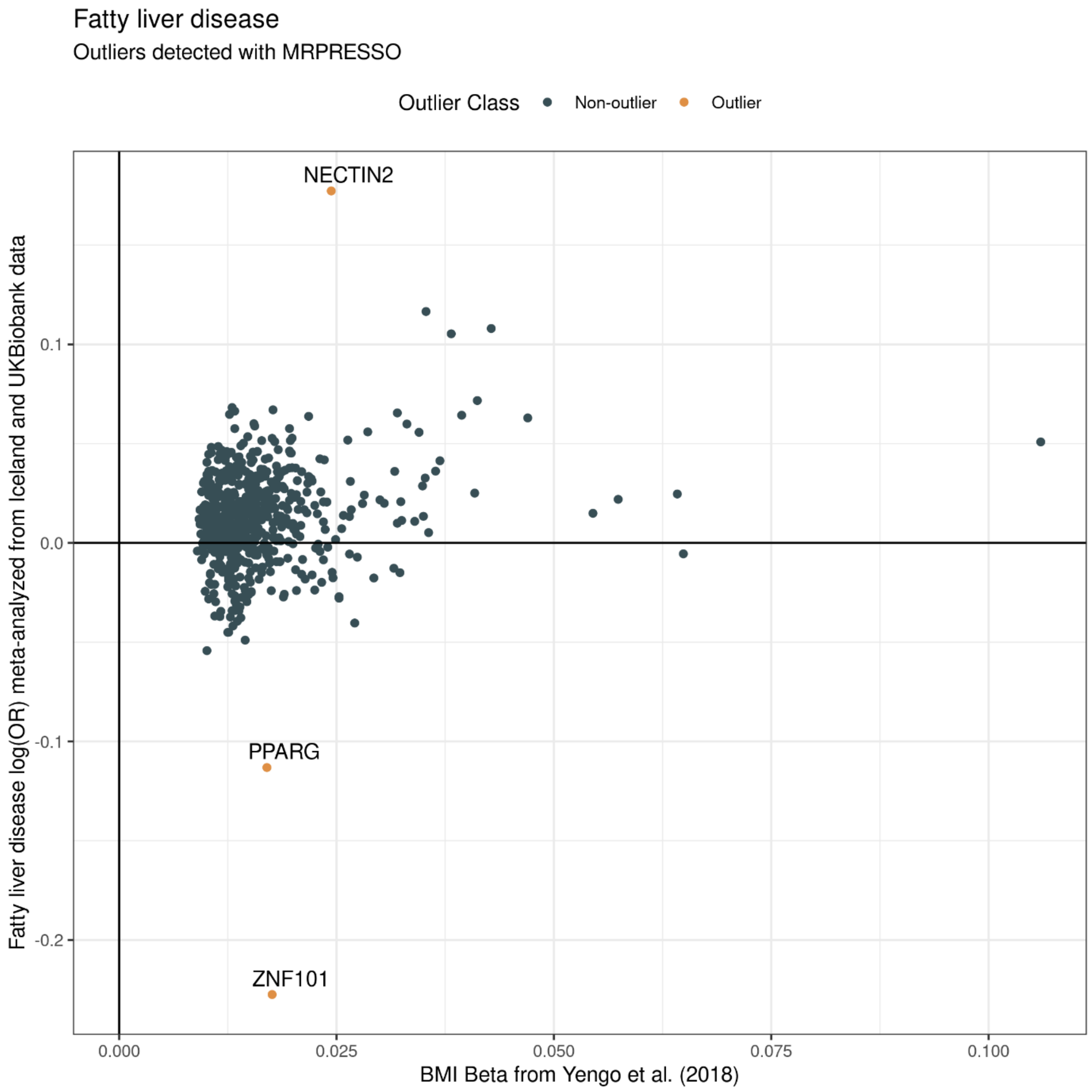

Supplementary Figure 3 Scatter plot showing effects of BMI variants on BMI on the x-axis and their effects (log(OR)) on fatty liver disease on the y-axis. Outliers detected with MRPRESSO are colored in orange and annotated with the closest gene.

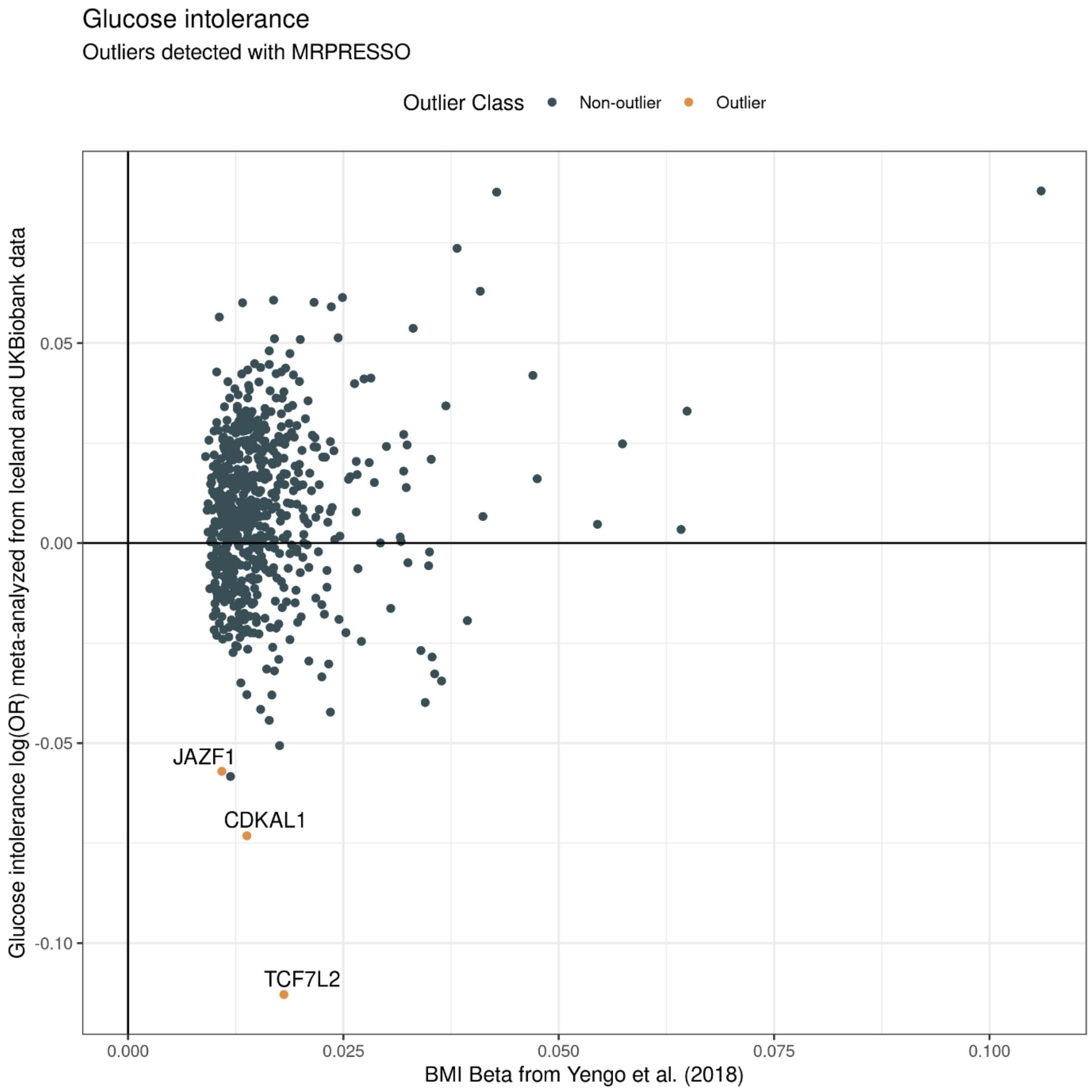

Supplementary Figure 4 Scatter plot showing effects of BMI variants on BMI on the x-axis and their effects (log(OR)) on glucose intolerance on the y-axis. Outliers detected with MRPRESSO are colored in orange and annotated with the closest gene.

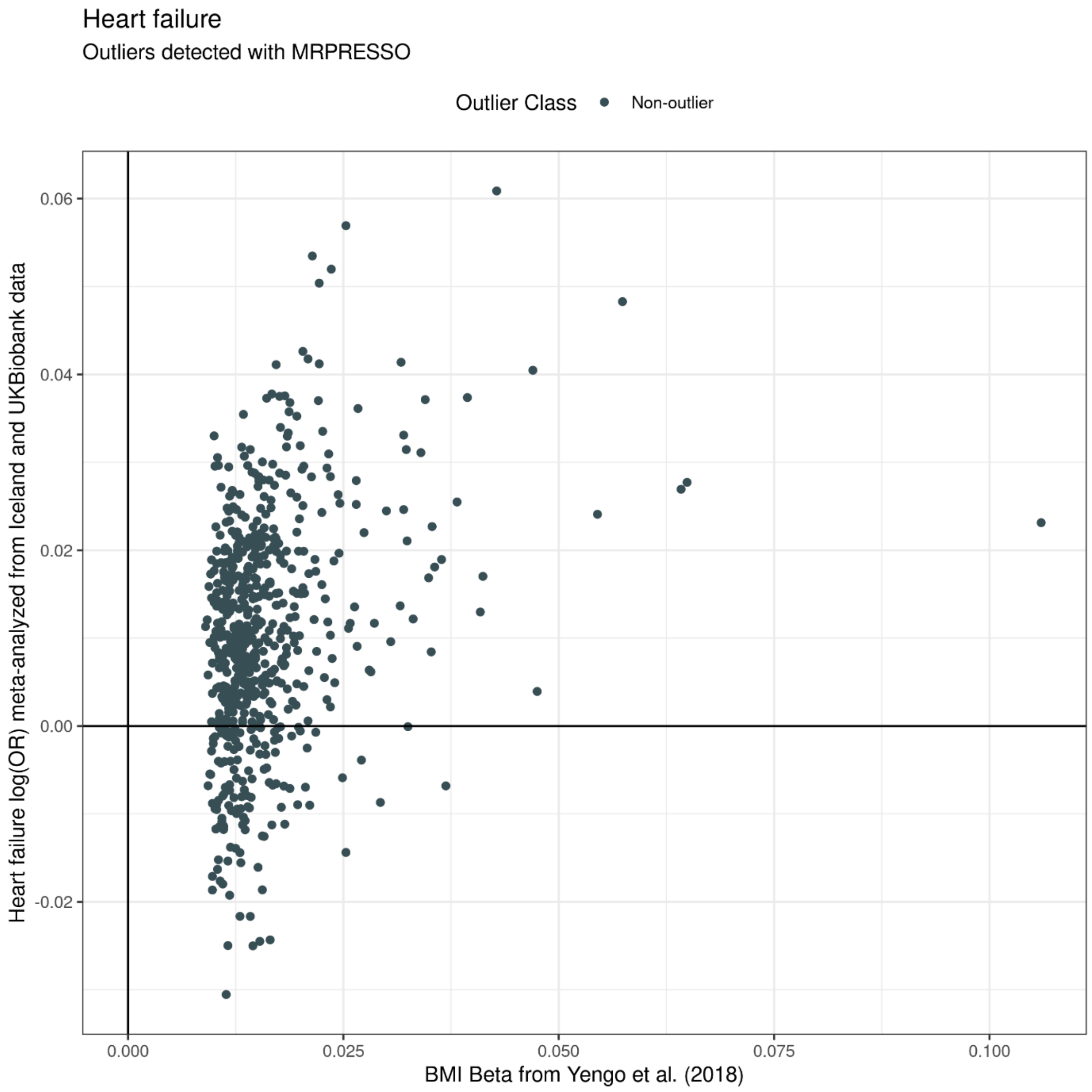

Supplementary Figure 5 Scatter plot showing effects of BMI variants on BMI on the x-axis and their effects (log(OR)) on heart failure on the y-axis. Outliers detected with MRPRESSO are colored in orange and annotated with the closest gene.

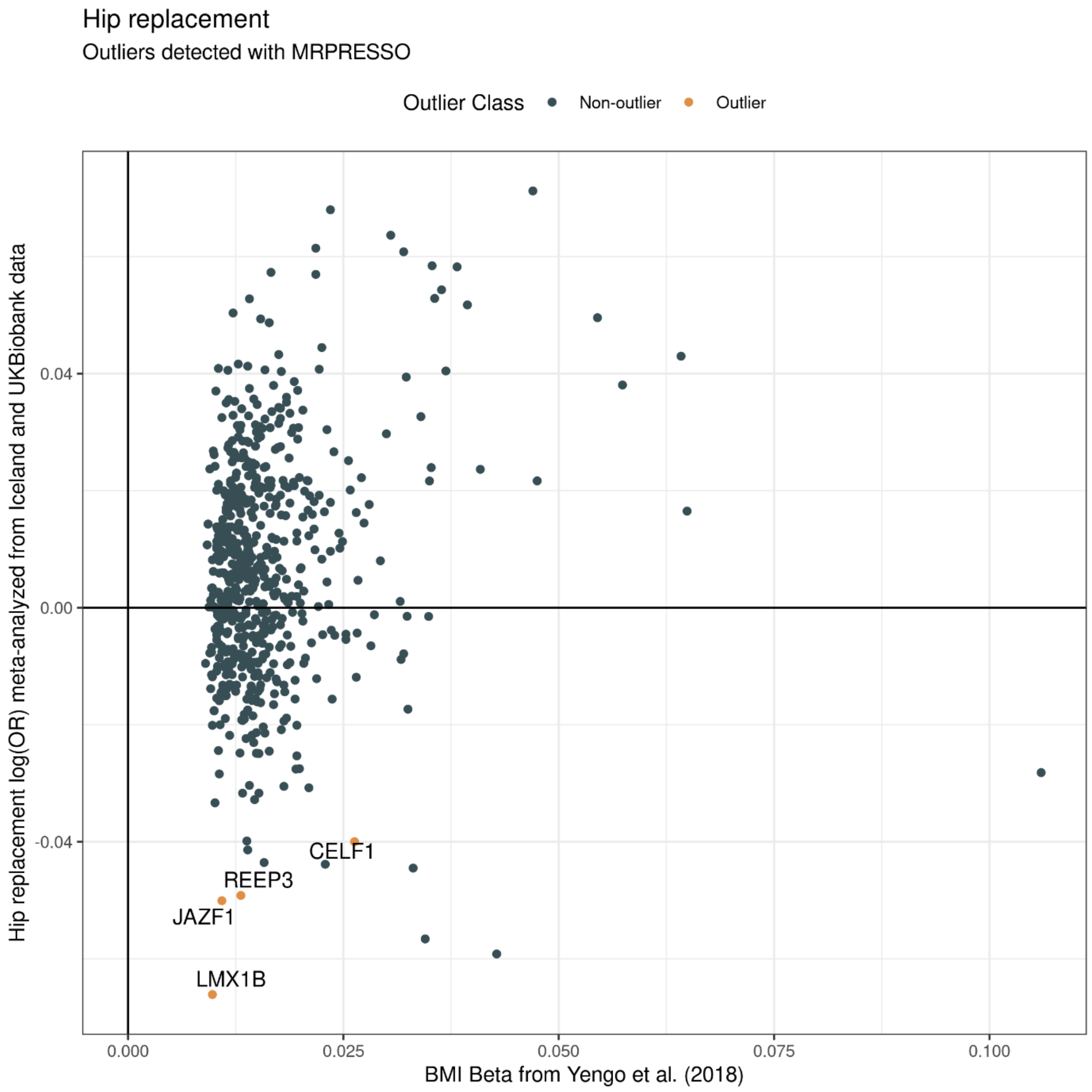

Supplementary Figure 6 Scatter plot showing effects of BMI variants on BMI on the x-axis and their effects (log(OR)) on hip replacement on the y-axis. Outliers detected with MRPRESSO are colored in orange and annotated with the closest gene.

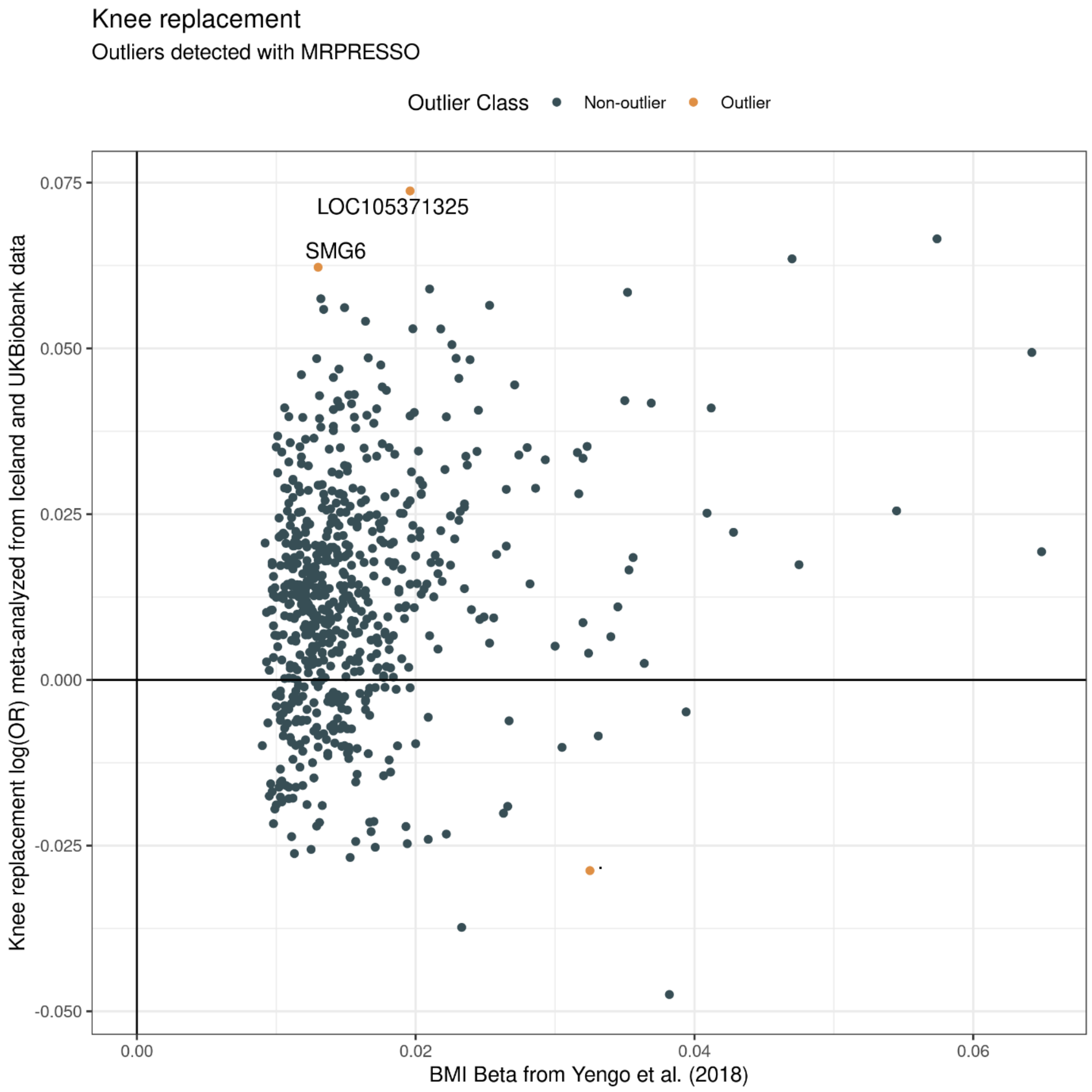

Supplementary Figure 7 Scatter plot showing effects of BMI variants on BMI on the x-axis and their effects (log(OR)) on knee replacement on the y-axis. Outliers detected with MRPRESSO are colored in orange and annotated with the closest gene.

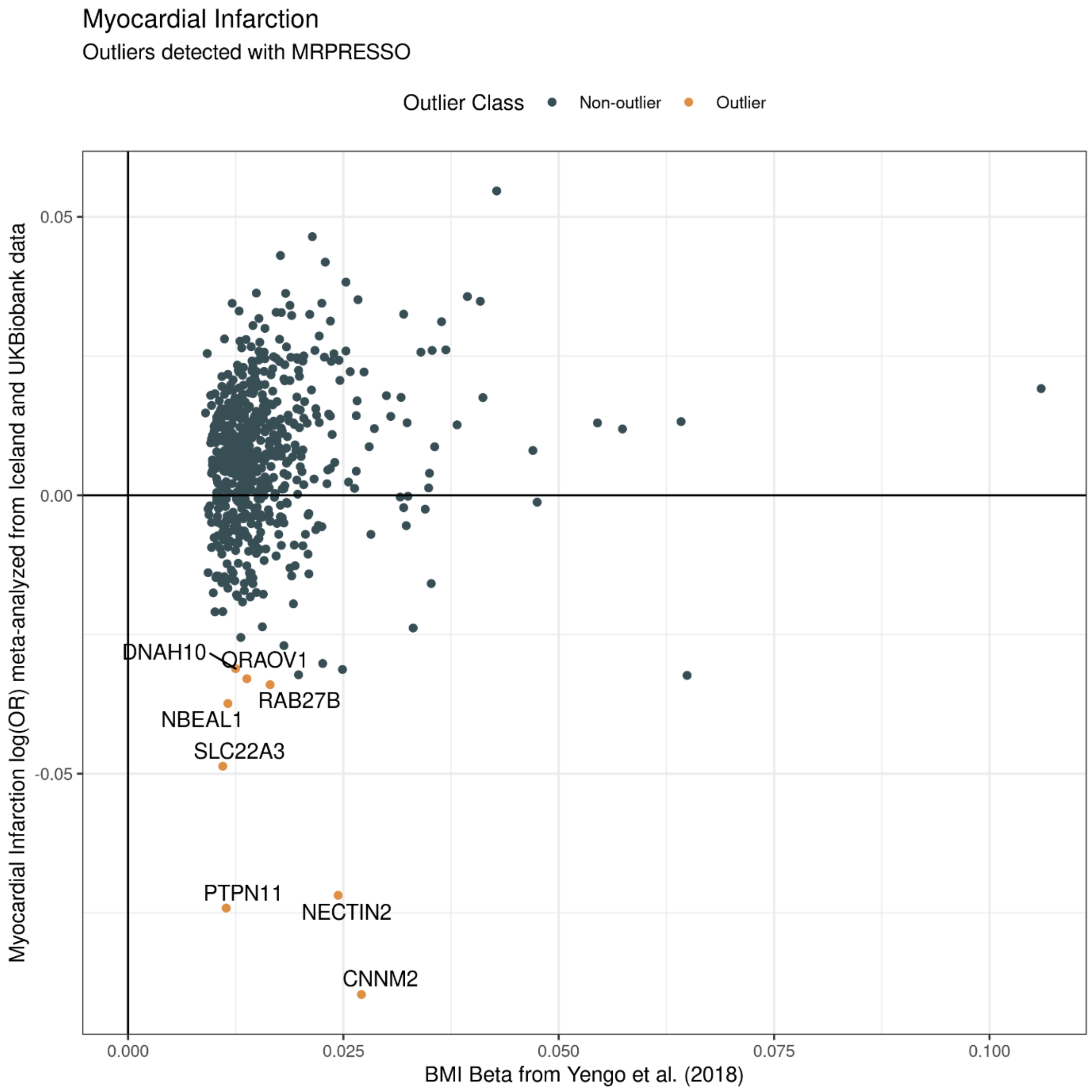

Supplementary Figure 8 Scatter plot showing effects of BMI variants on BMI on the x-axis and their effects (log(OR)) on myocardial infarction on the y-axis. Outliers detected with MRPRESSO are colored in orange and annotated with the closest gene.

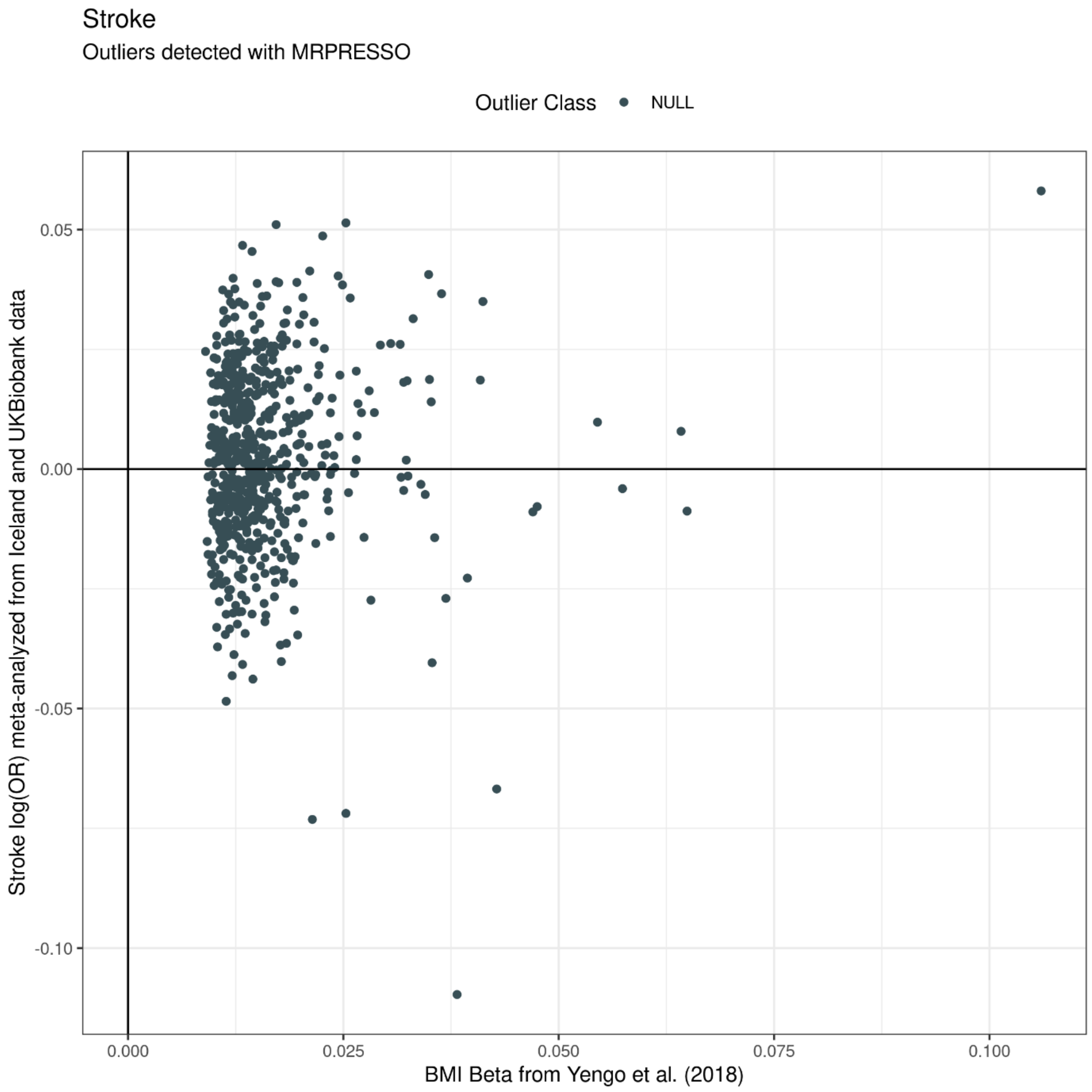

Supplementary Figure 9 Scatter plot showing effects of BMI variants on BMI on the x-axis and their effects (log(OR)) on stroke on the y-axis. Outliers detected with MRPRESSO are colored in orange and annotated with the closest gene.

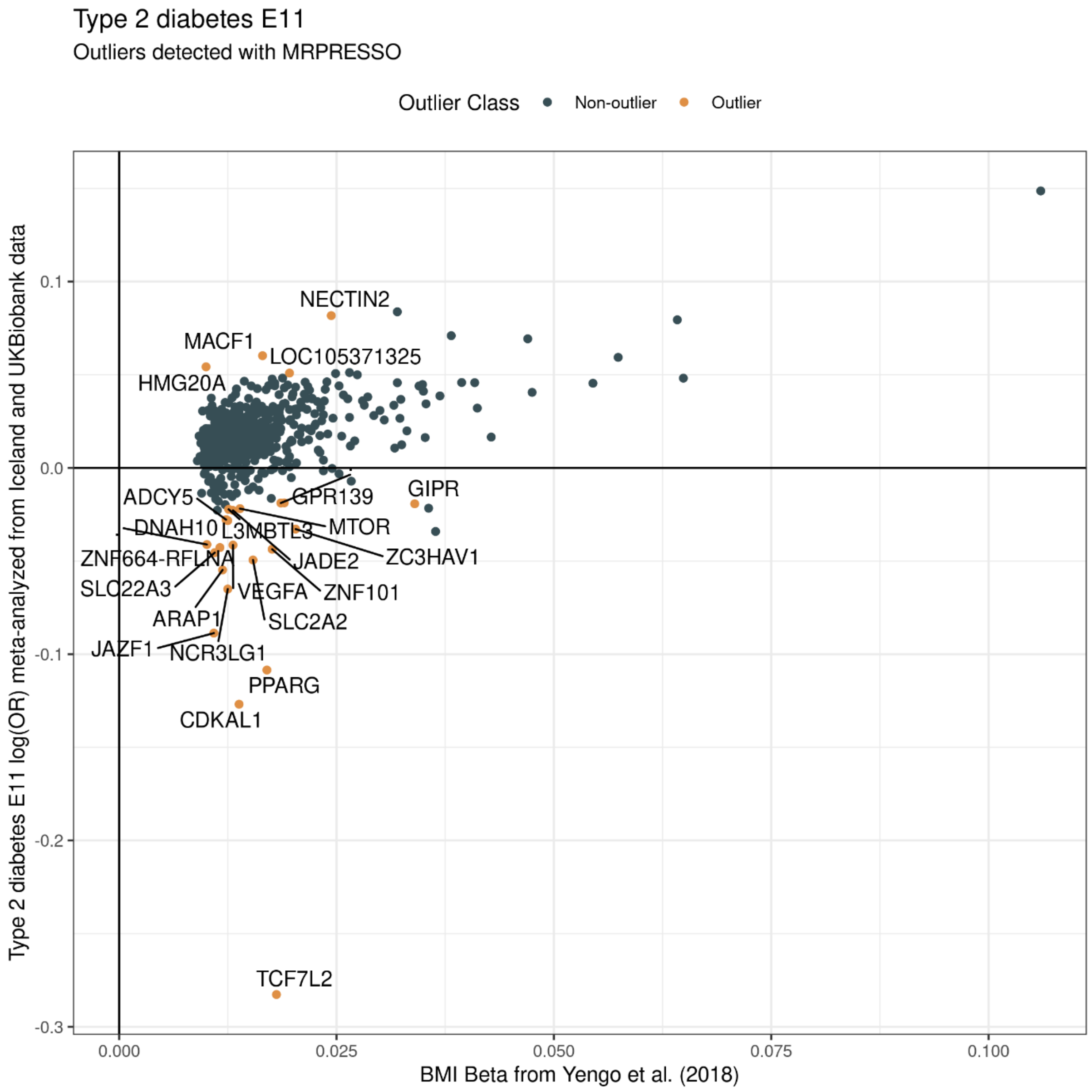

Supplementary Figure 10 Scatter plot showing effects of BMI variants on BMI on the x-axis and their effects (log(OR)) on type 2 diabetes on the y-axis. Outliers detected with MRPRESSO are colored in orange and annotated with the closest gene.

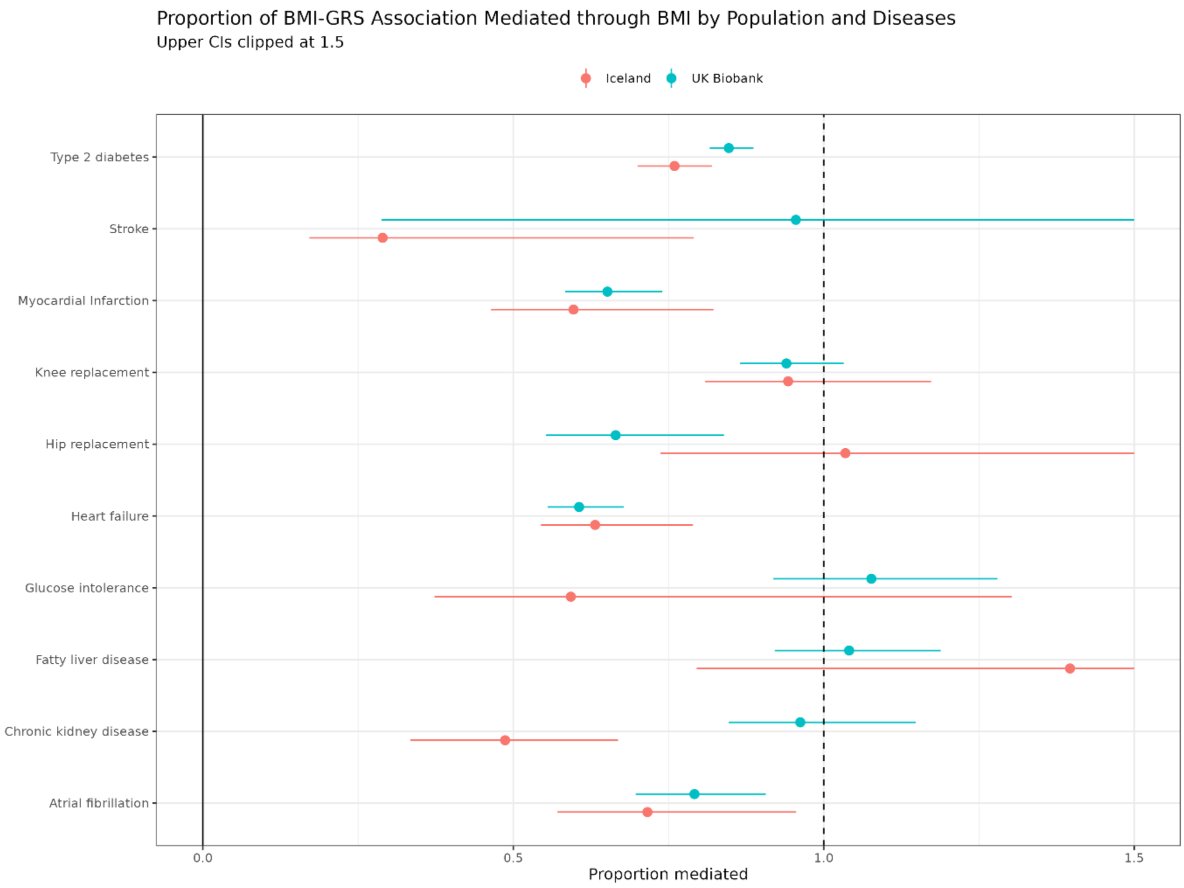

Supplementary Figure 11 Proportion of BMI-GRS association mediated by BMI itself. We compare these values between cohorts, values estimated in Icelandic data in red, and values estimated from UK data in blue. The confidence intervals are created by running the mediate function from the R package mediation, with the quasi-Bayesian method using 200 simulations. The underlying data is in supplementary table 4.

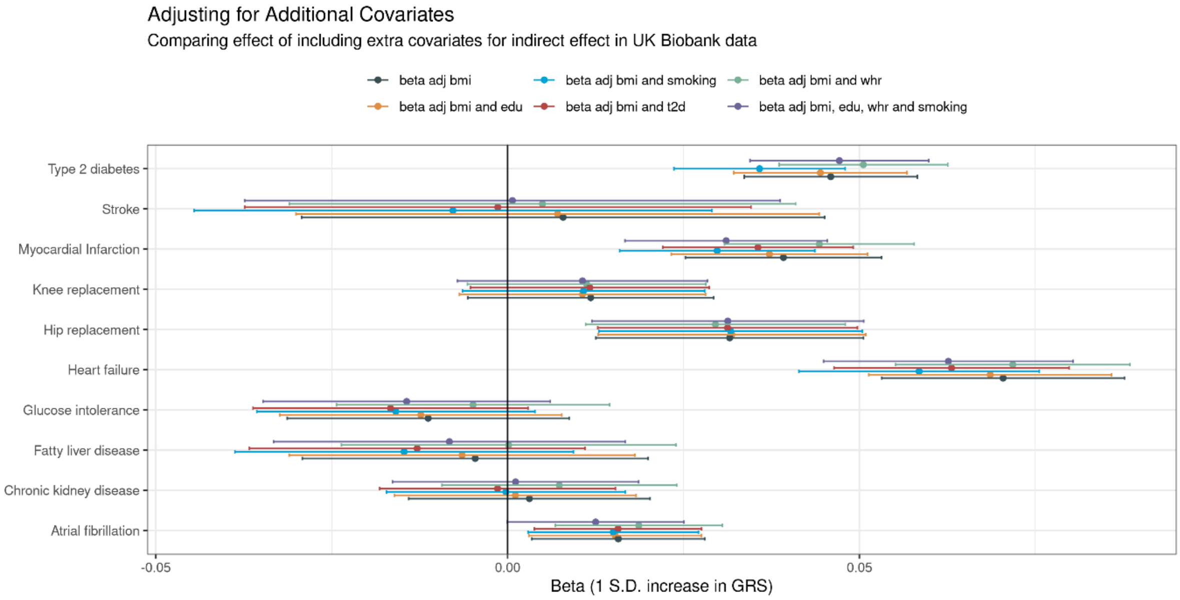

Supplementary Figure 12 Comparing effects of the BMI-GRS on diseases/conditions when including measured BMI in the model in addition to other potential confounders. The effects have not been scaled, so the unit represents a standard deviation increase in the GRS. The points represent point estimates for the GRS from logistic regression and the error bars represent 95% confidence intervals.

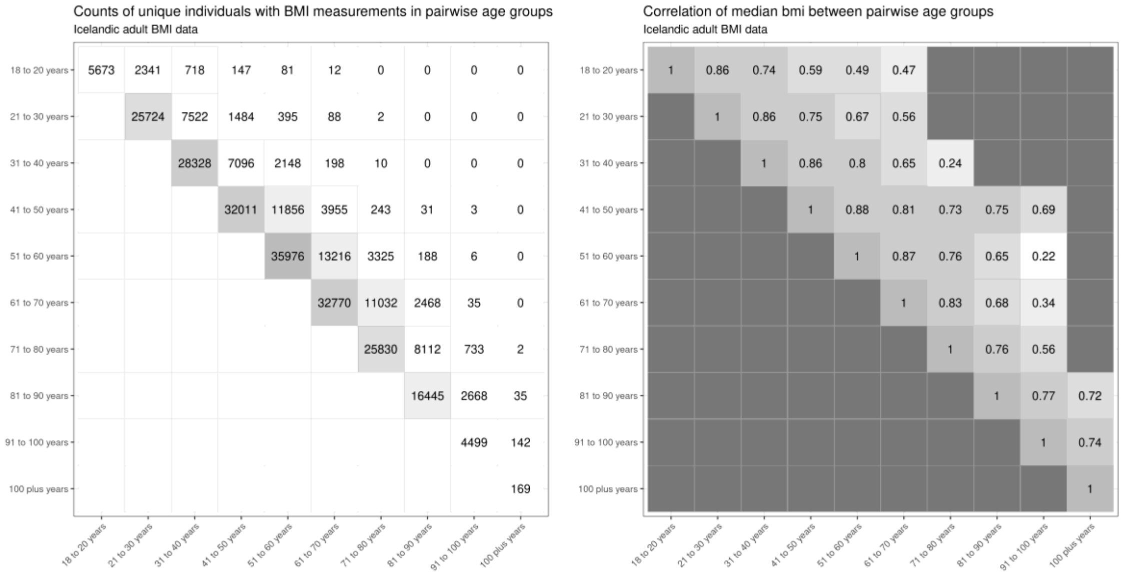

Supplementary Figure 13 Summary of repeated BMI measurements in the Icelandic dataset. On the left panel we quantify the number of individuals who have at least one BMI measurement according to different age strata, where each individual is only counted once in each unique block. On the right we have the correlation of the raw BMI measures for each individual, using the median in case of multiple measurements for the same individual within an age strata.

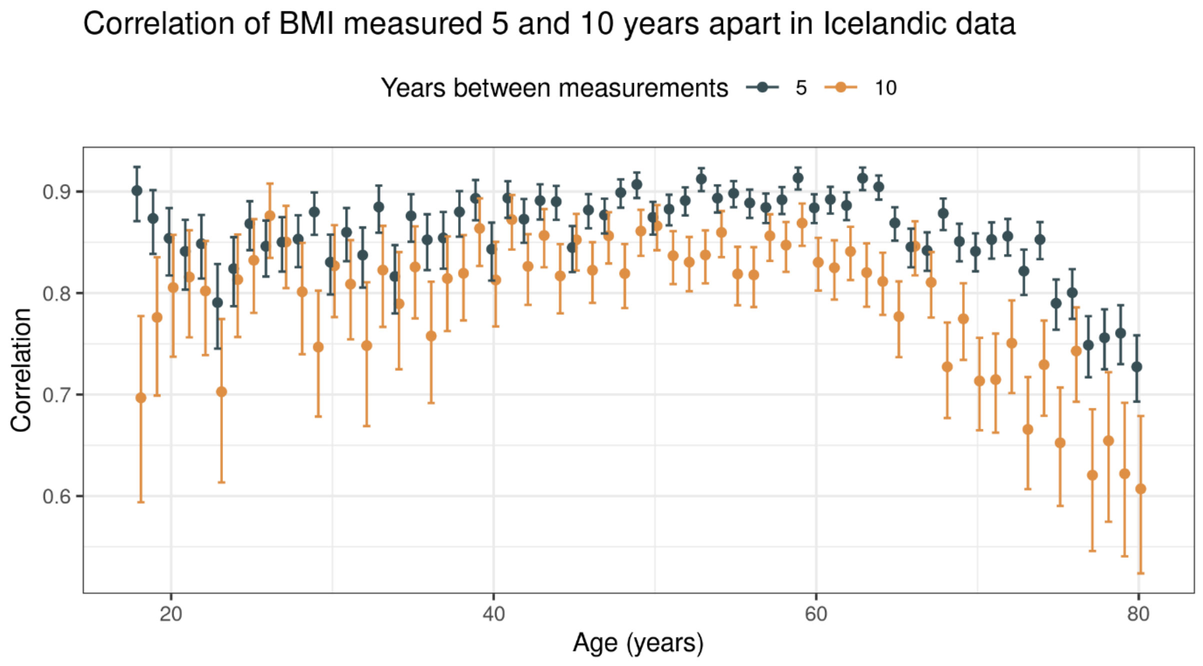

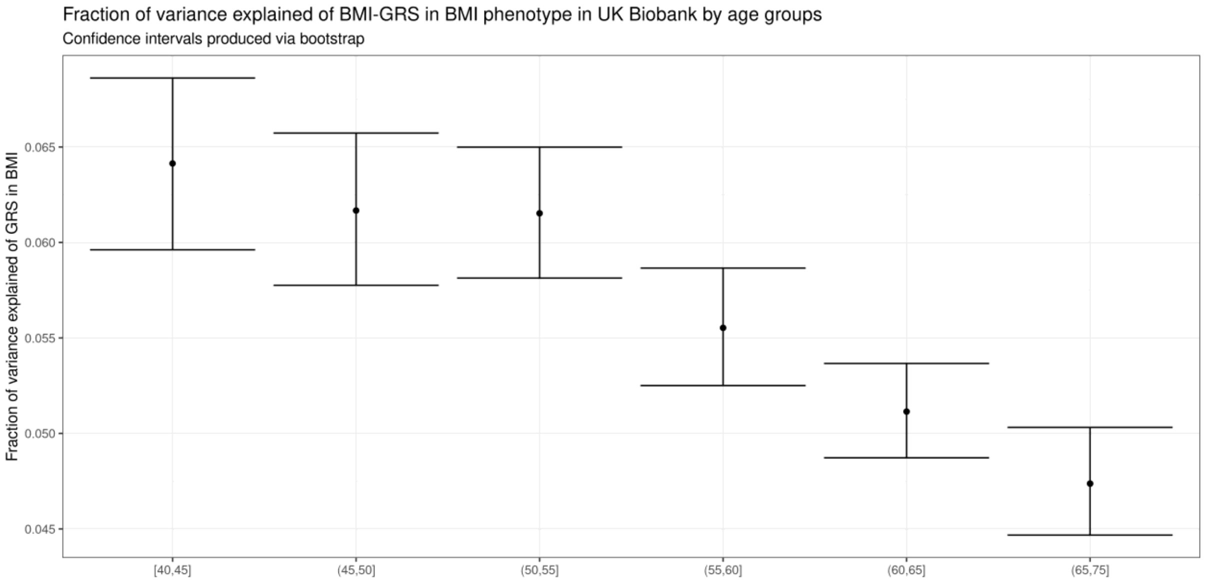

Supplementary Figure 14 Upper panel: Temporal Pearson correlations of BMI measurments in the Icelandic data with 95% confidence intervals. For each specific age, we looked for all individuals that had a BMI measurement exactly 5 and 10 years later, we subsequently computed the Pearson correlation of the BMI at the specific age, with the BMI 5 and 10 years later. In case of multiple measurements at the same age for an individual we used the median. We observe a decline in correlation after 60. Lower panel: Variance explained by the BMI-GRS without outliers in the UK Biobank BMI phenotype by age at recruitment groups, the points represent the median from a bootstrap, and the error bars represent a 95% bootstrap confidence interval.

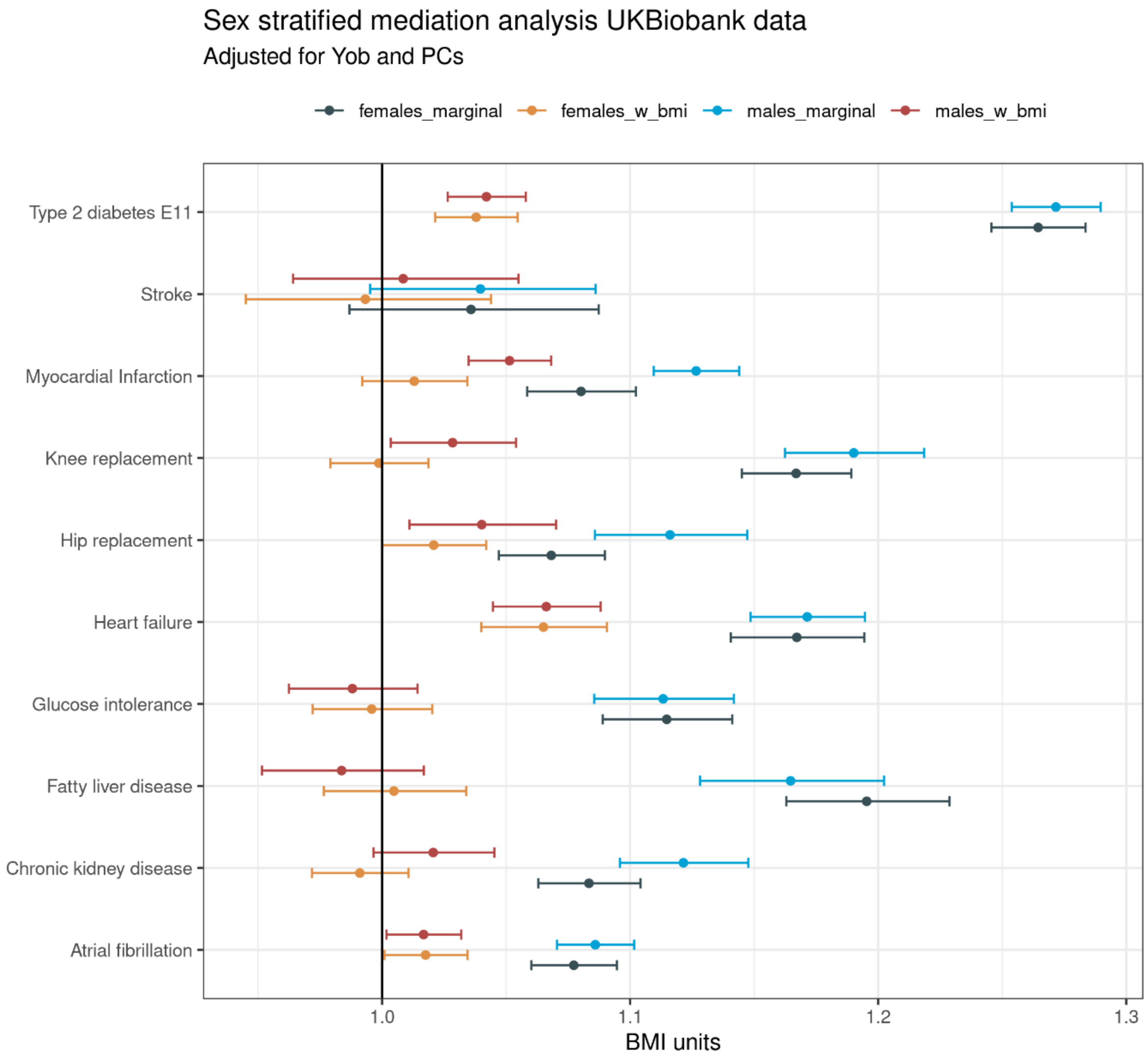

Supplementary Figure 15 Main association results from the UK stratified by sex. The BMI-GRS (score without outliers) is scaled specifically within each sex such that an increase by one unit corresponded to a one unit (kg/m^2) increase in BMI, thus the ORs correspond to a 1 unit increase in BMI (Supplementary Table 5b). The blue and black points and error-bars show the disease association of the BMI-GRS adjusted for year of birth and 20 genetic principal components, the orange and red points and error-bars show the corresponding association when BMI has been added as a covariate. The error bars correspond to a 95% confidence interval for the parameter and the points represent ORs estimated with logistic regression. See supplementary table 5a for details.

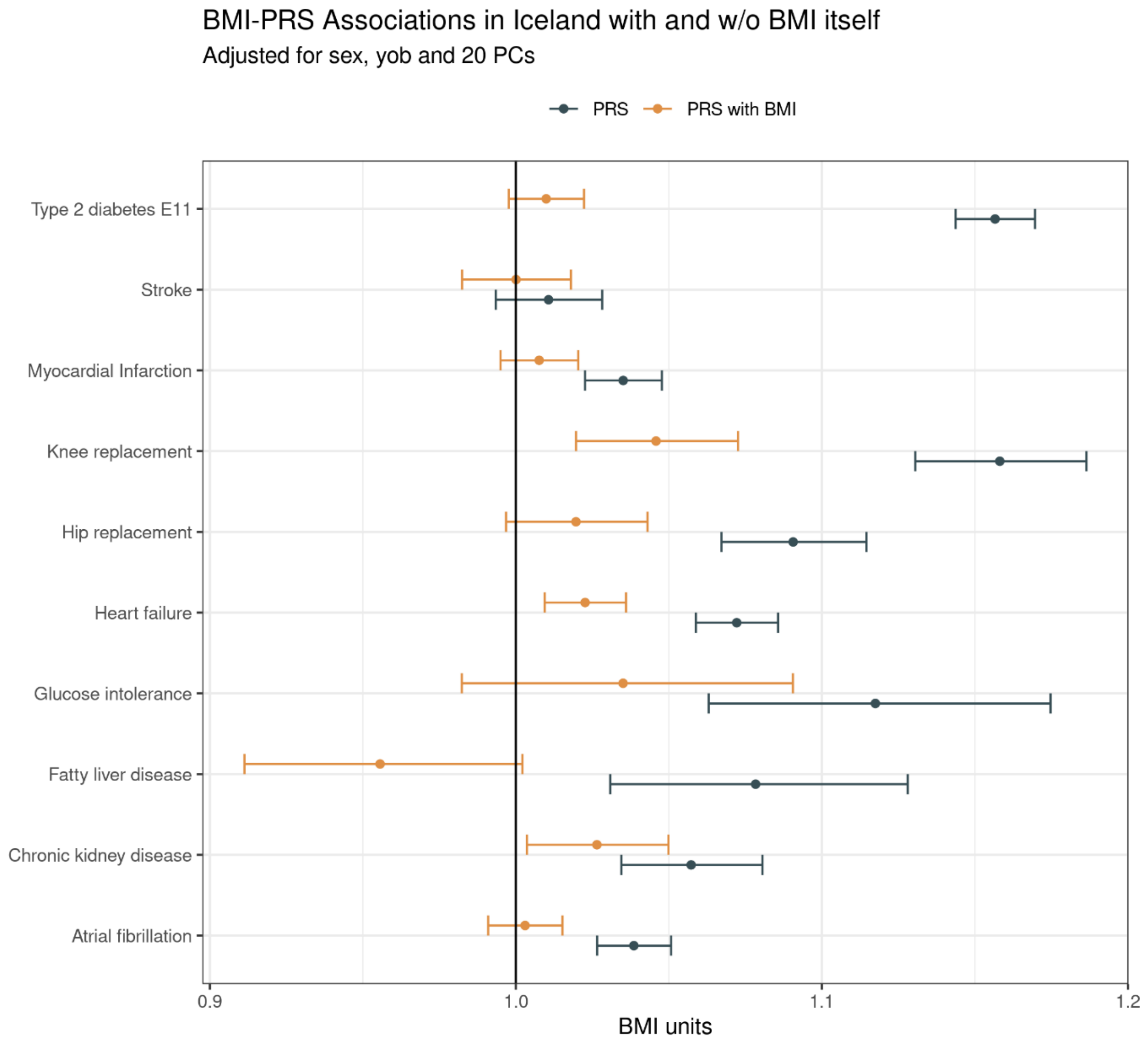

Supplementary Figure 16 Mediation analysis repeated in the Icelandic data using the PRS a liberal score made with 611K markers. The error bars correspond to a 95% confidence interval for the parameter and the points represent ORs from logistic regression.

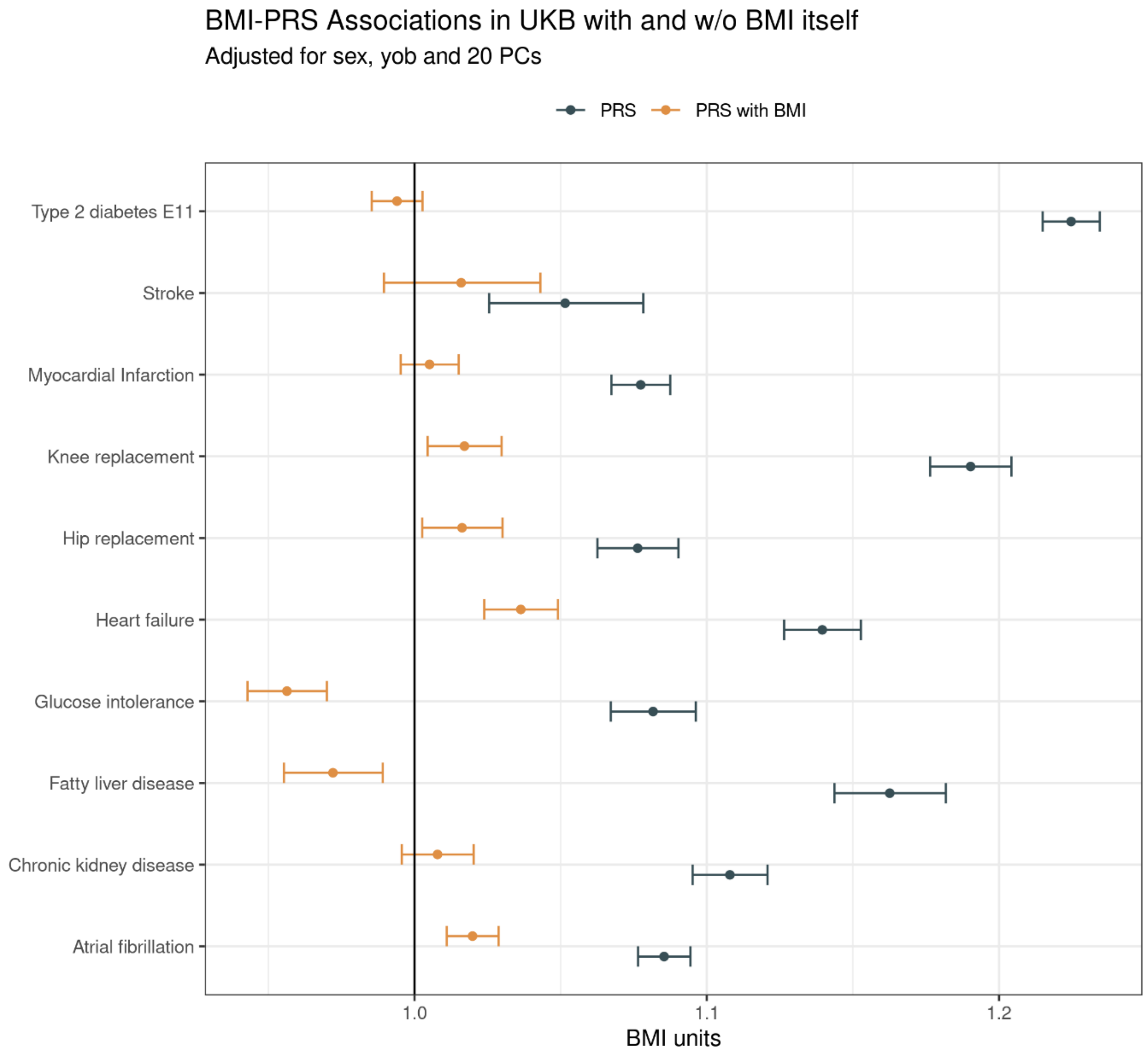

Supplementary Figure 17 Mediation analysis repeated in the UK data using the PRS a liberal score made with 611K markers. The error bars correspond to a 95% confidence interval for the parameter and the points represent ORs from logistic regression.

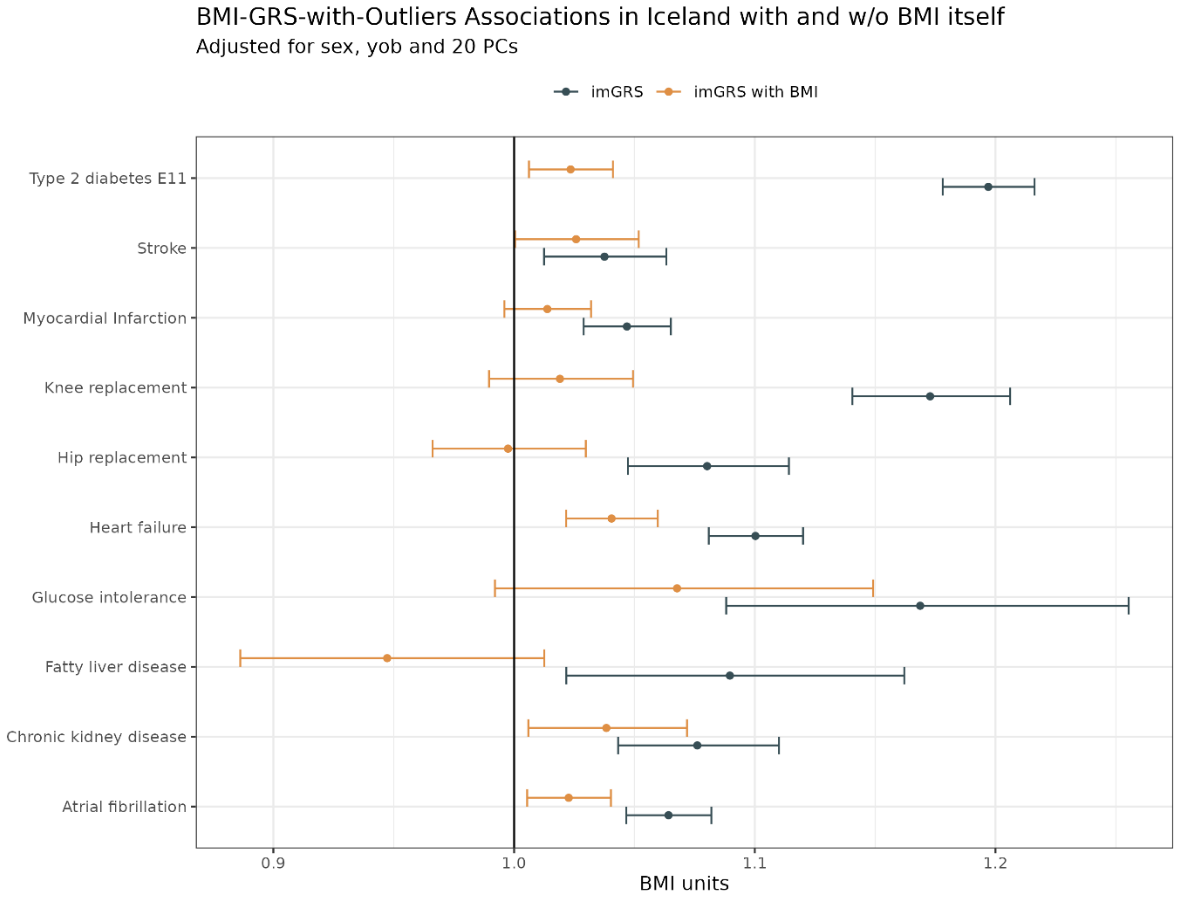

Supplementary Figure 18 Mediation analysis repeated in the Icelandic data using the BMI-GRS-with-Outliers which consists of all independent markers (liberal GRS score and outliers). The error bars correspond to a 95% confidence interval for the parameter and the points represent ORs from logistic regression.

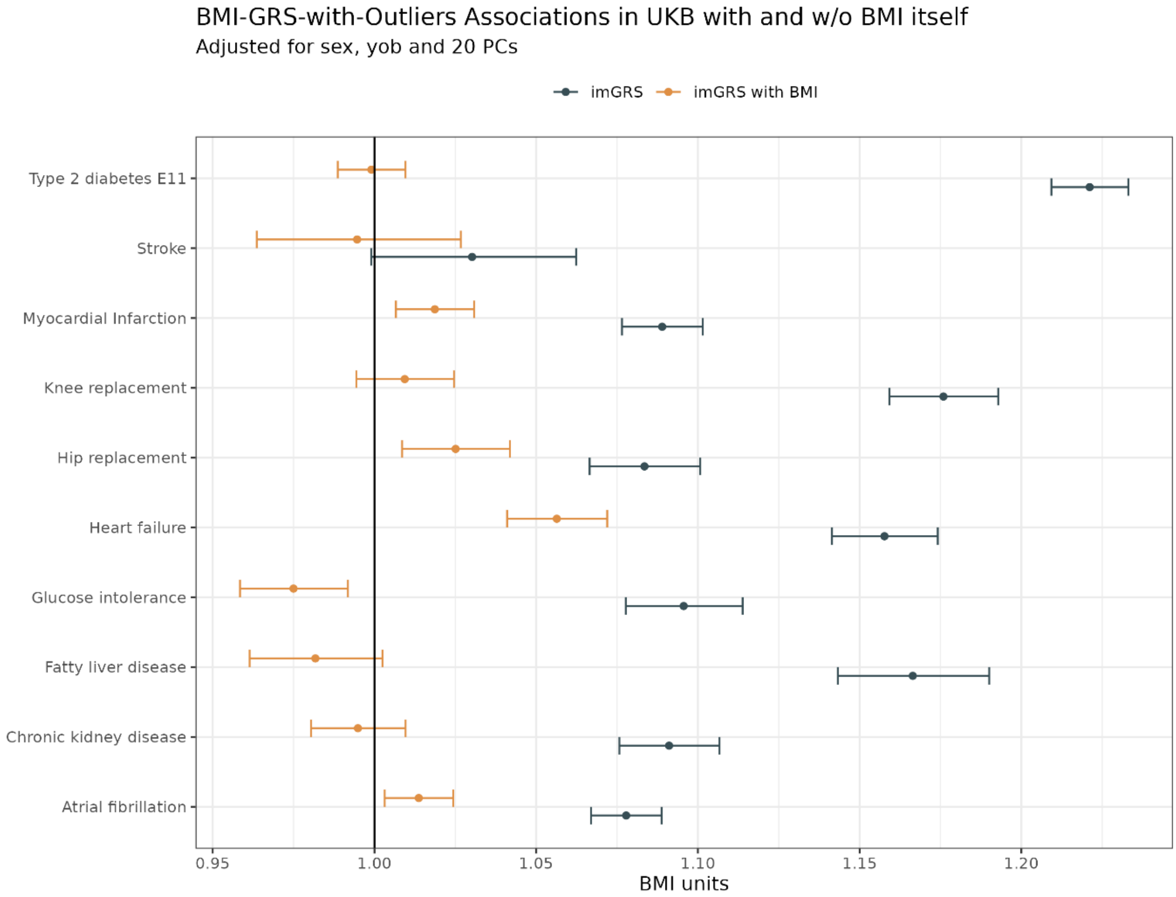

Supplementary Figure 19 Mediation analysis repeated in the UK data using the BMI-GRS-with-Outliers which consists of all independent markers (liberal GRS score and outliers). The error bars correspond to a 95% confidence interval for the parameter and the points represent ORs from logistic regression.

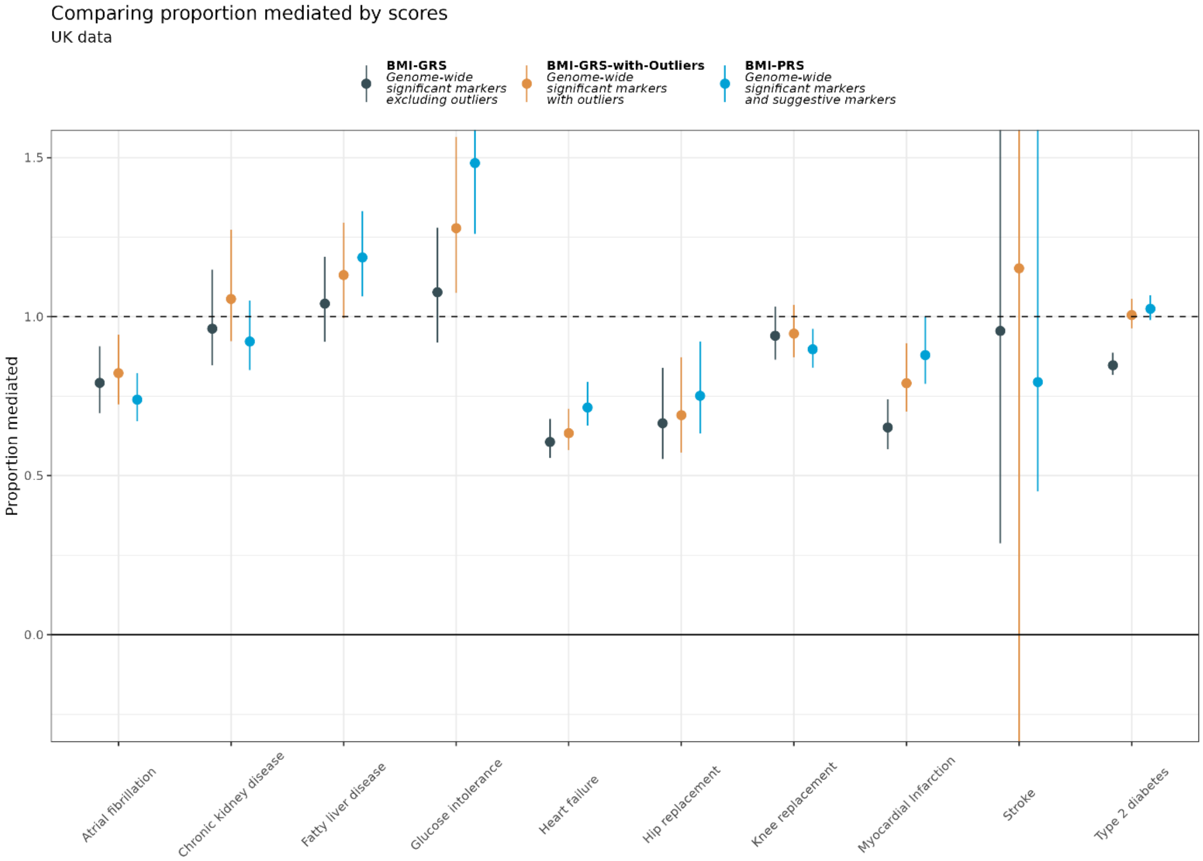

Supplementary Figure 20 Proportion mediated by BMI for the different scores in UK data (Supplementary Table 4).

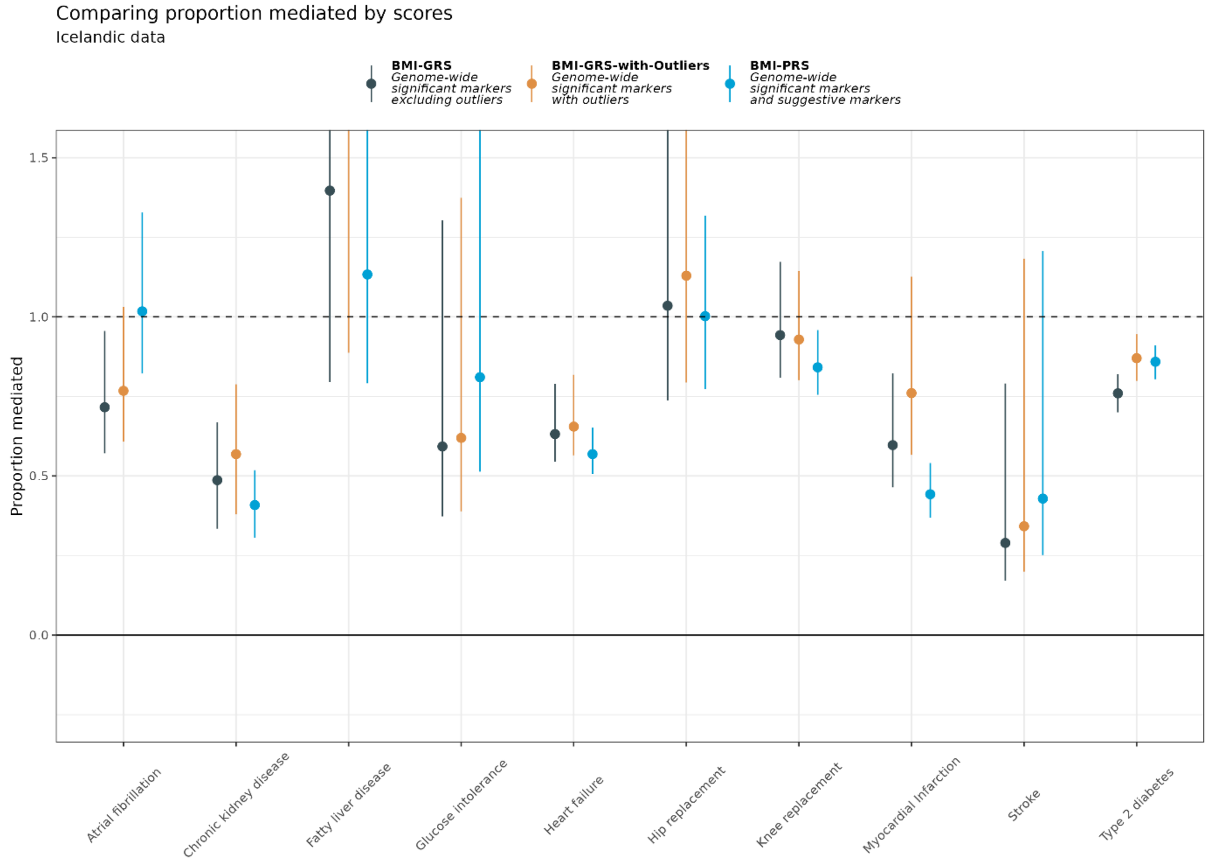

Supplementary Figure 21 Proportion mediated by BMI for the different scores in Icelandic data (Supplementary Table 4).

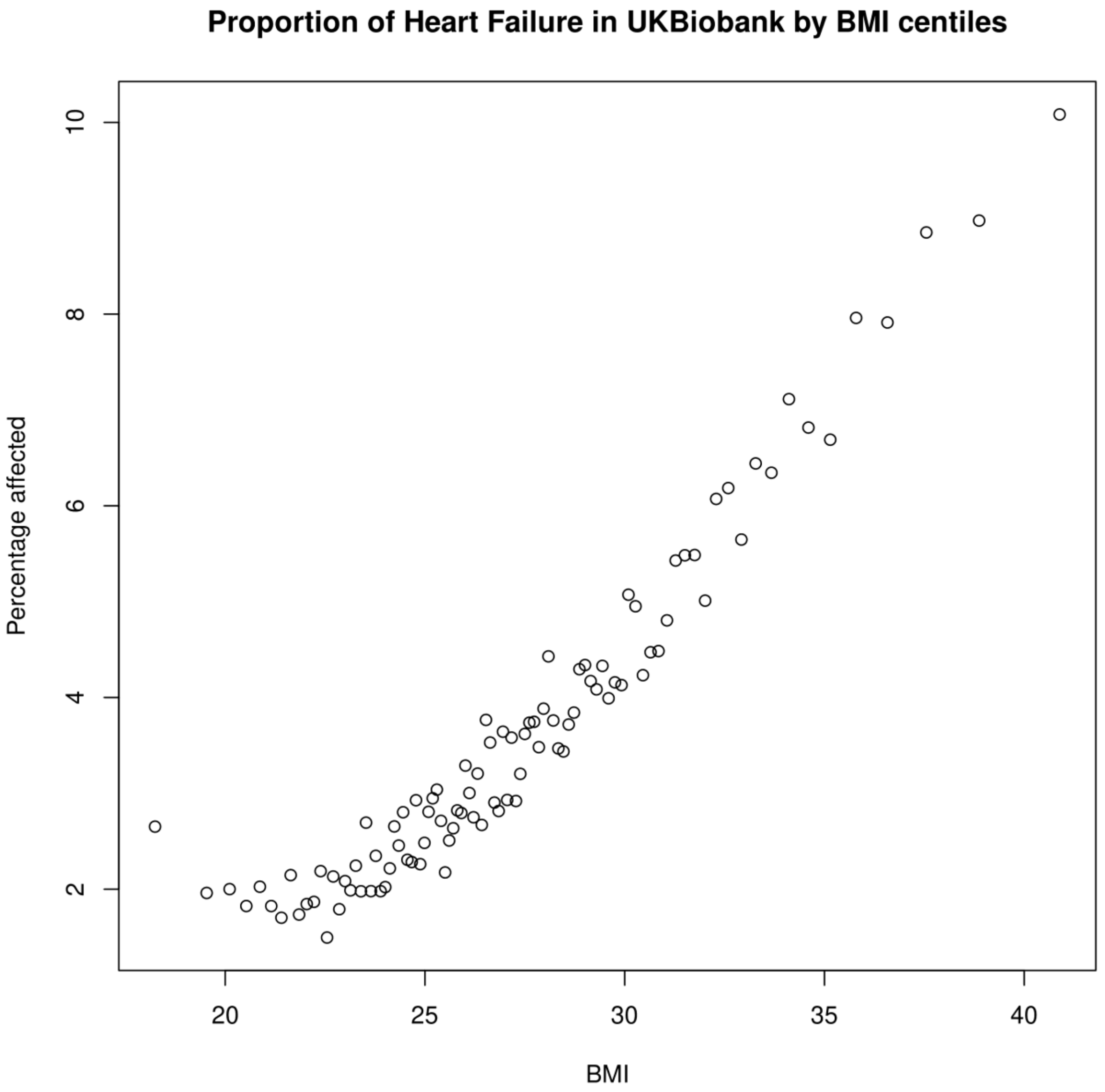

Supplementary Figure 22 Proportion of individuals diagnosed with heart failure by BMI percentile, where BMI is the BMI at first visit to the UK Biobank.

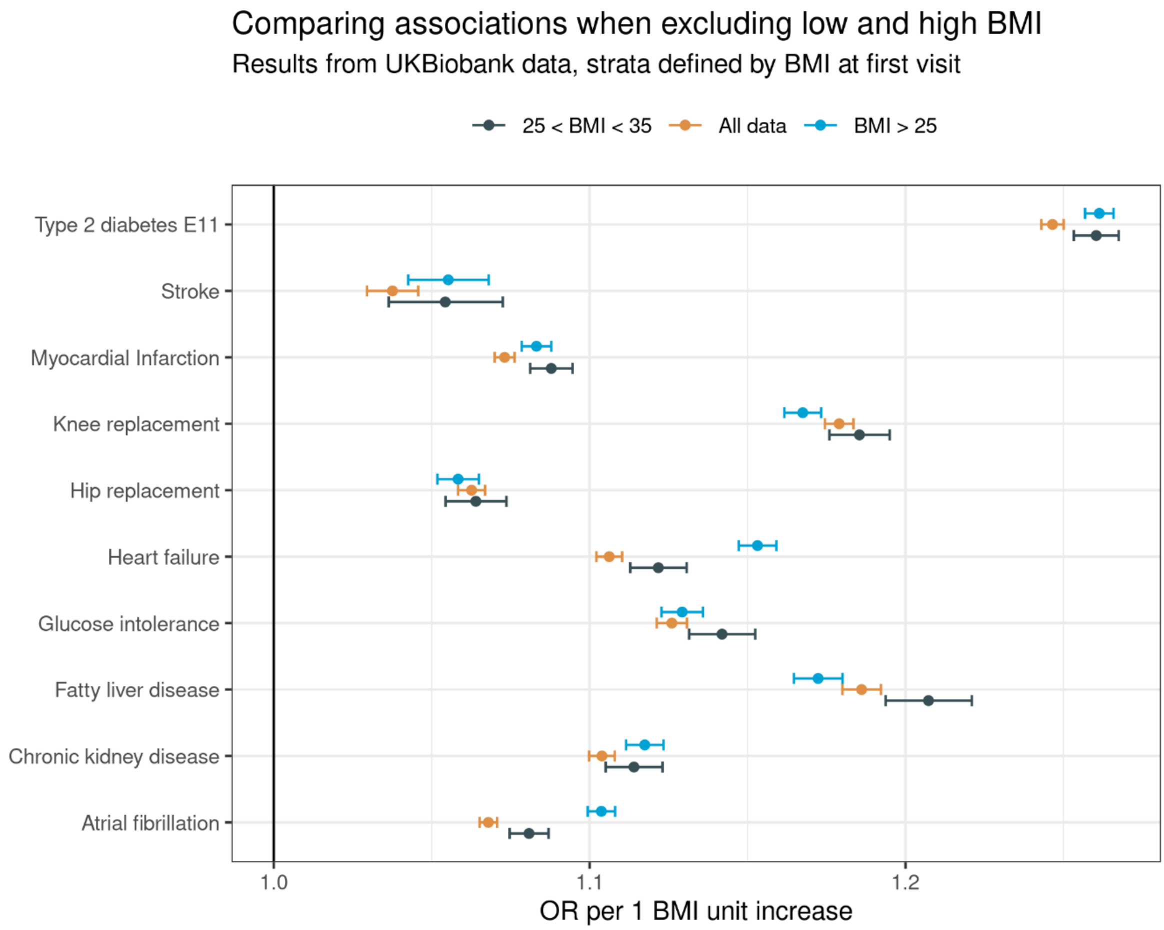

Supplementary Figure 23 Association of BMI Phenotype when restricting to specific BMI ranges in the UK Biobank data. The orange line corresponds to all data, whereas the blue one is excluding BMI below 25 and the black one is only individuals with BMI between 25 and 35. The error bars correspond to a 95% confidence interval for the parameter and the points represent ORs from logistic regression

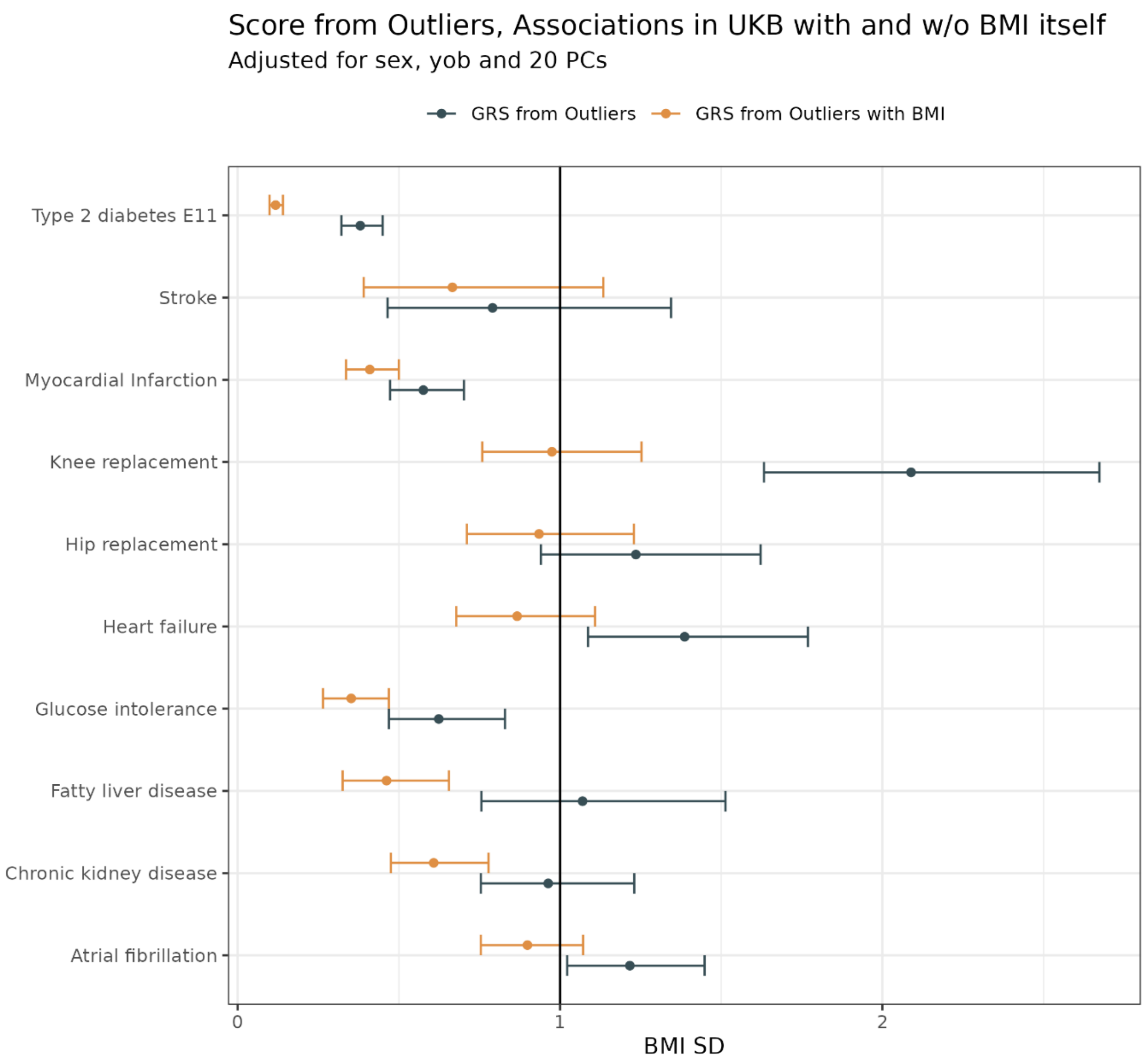

Supplementary Figure 24 Mediation analysis results for score created from outlier variants in UK data. The error bars correspond to a 95% confidence interval for the parameter and the points represent ORs from logistic regression

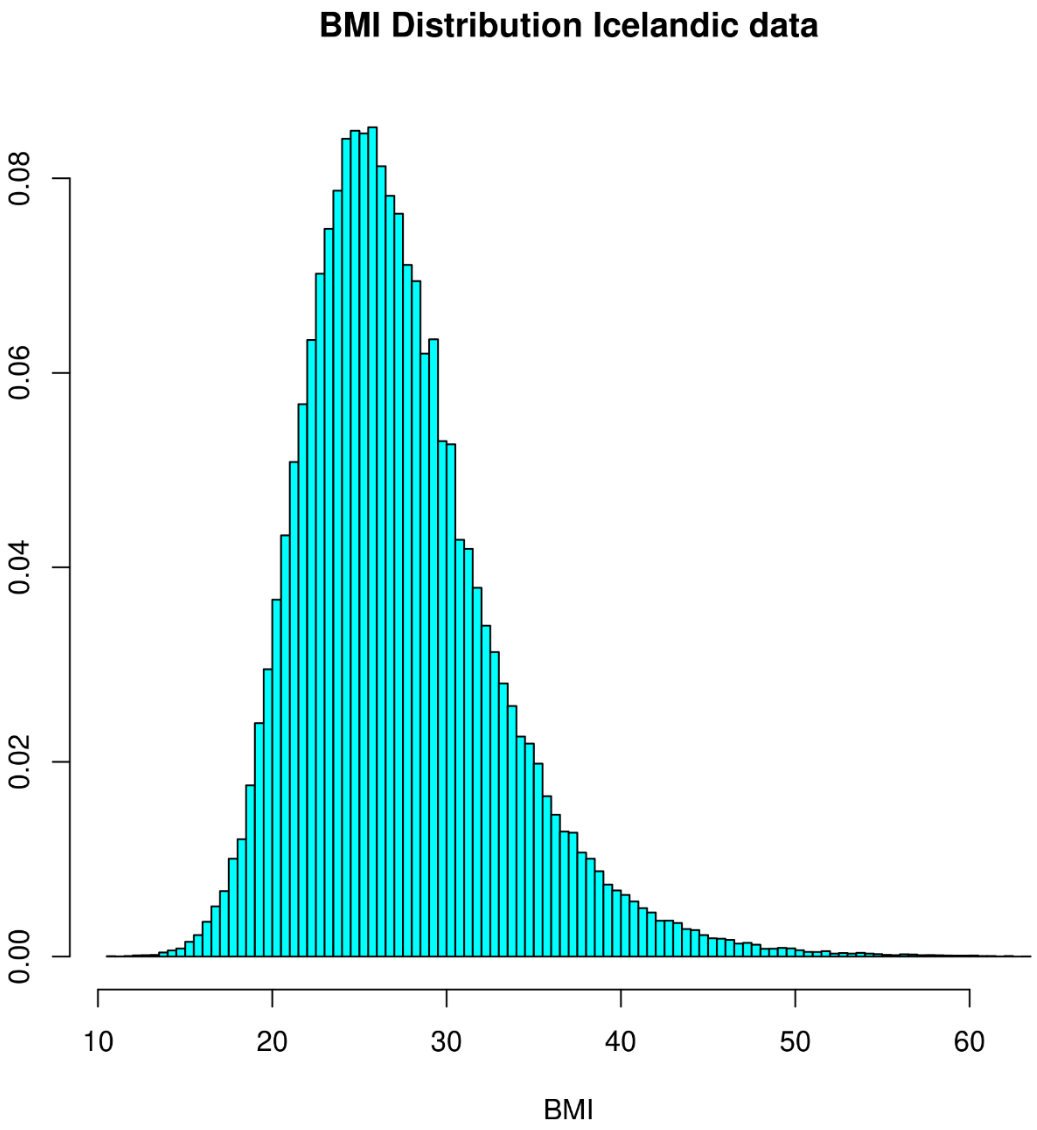

Supplementary Figure 25 Distribution of raw BMI measurements in icelandic data. For individuals with multiple measurements, the value is averaged.

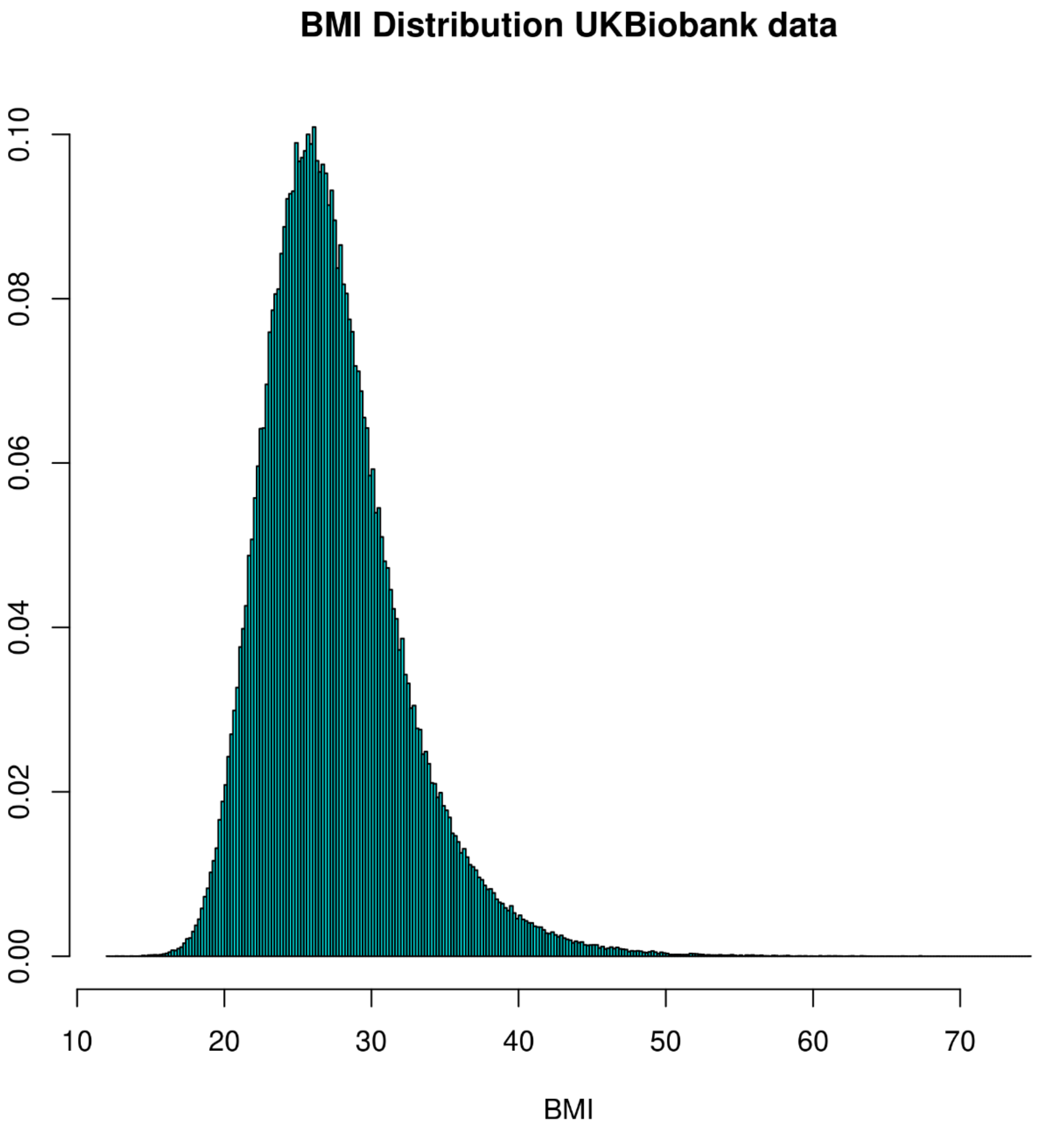

Supplementary Figure 26 Distribution of BMI at first visit in the UK Biobank data.

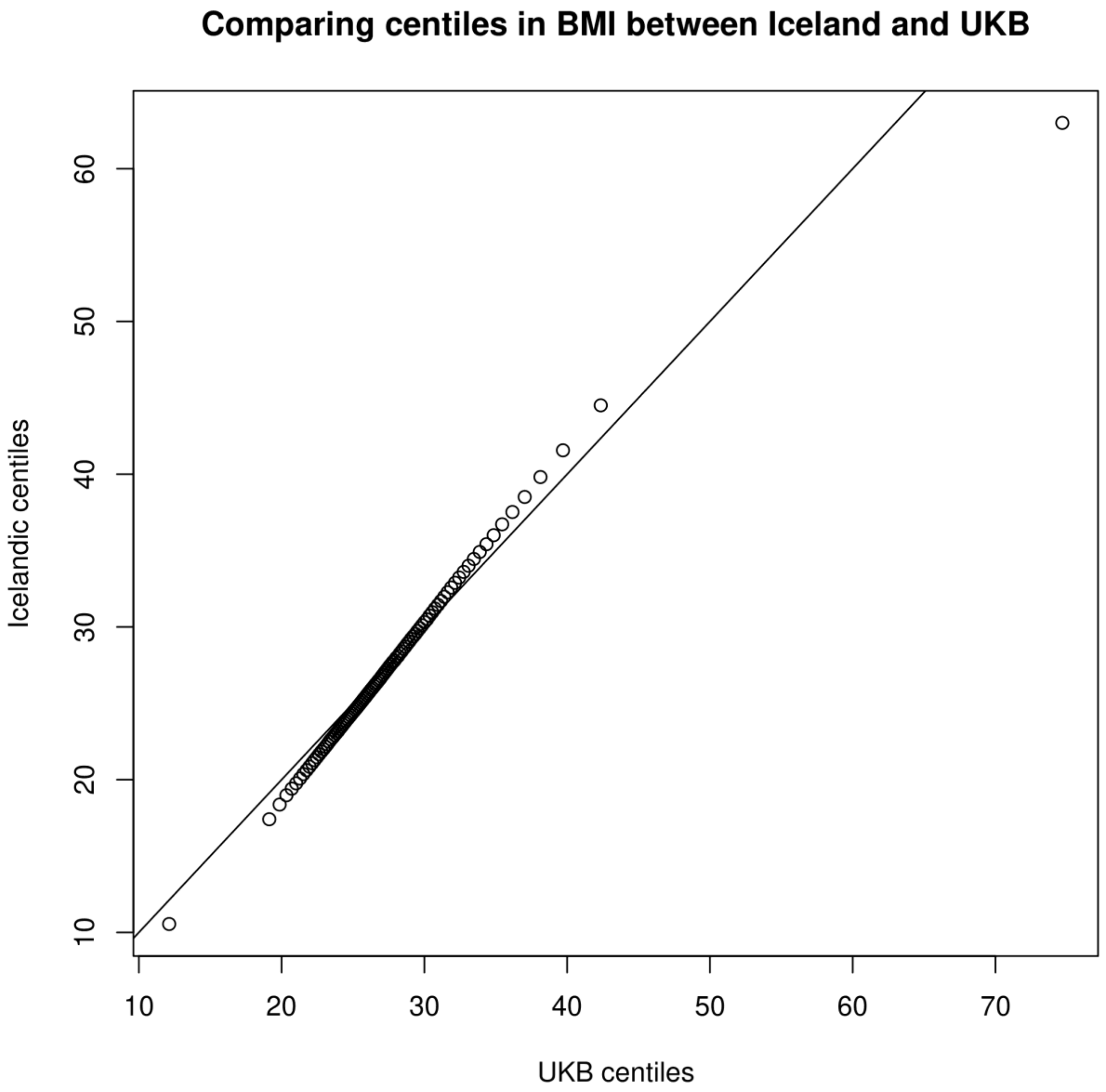

Supplementary Figure 27 Comparing distribution of BMI in Iceland and UKBiobank. More spread is observed in the Icelandic data. The highest centile is much larger in the UKBiobank data.

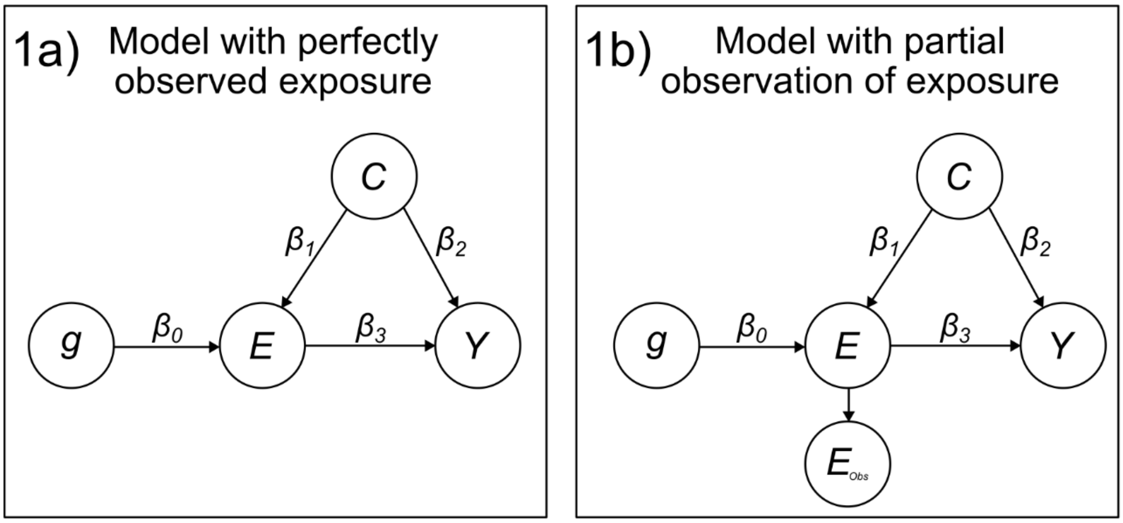

Supplementary Figure 28 On the left (panel 1a)) we have a model with perfect observation of the exposure, on the right (panel 1b)) we have a node depicting partial observation of the exposure.

Overview of supplementary tables

S1	Associations of BMI GRSs and PRSs with the BMI phenotype and raw BMI measurements
S2	Summary of BMI measurements
S3	Main mediation results with the BMI-GRS score (outliers excluded)
S4	Mediation results by sex in UK data with the BMI-GRS score (outliers excluded)
S5	Association of BMI-GRS with raw BMI by sex
S6	Mediation analysis with BMI-GRS-with-Outliers
S7	Mediation analysis with BMI-PRS
S8	Proportion Mediated by Scores
S9	Association of BMI phenotype with outcomes
S10	Association of BMI phenotype with outcomes stratified by BMI at first visit to UK Biobank

Associations of BMI-GRSs and PRSs with the BMI phenotype and raw BMI measurements

score	Outcome	N	beta score	score SE	-log10 (p)	R2
UKB_PRS	BMI phenotype	429700	0.298	0.0015	9059.9	0.0885
UKB_GRS-with-Outliers	BMI phenotype	429700	0.243	0.0015	5855.5	0.0590
UKB_GRS	BMI phenotype	429700	0.235	0.0015	5455.8	0.0552
Iceland_PRS	BMI phenotype	101304	0.319	0.0028	2815.6	0.1076
Iceland_GRS-with-Outliers	BMI phenotype	101304	0.222	0.0029	1303.6	0.0530
Iceland_GRS	BMI phenotype	101304	0.213	0.0029	1201.2	0.0491
UKB_PRS	BMI Raw	429628	1.393	0.0069	8802.7	0.0852
UKB_GRS-with-Outliers	BMI Raw	429628	1.136	0.0070	5675.8	0.0566
UKB_GRS	BMI Raw	429628	1.099	0.0070	5282.3	0.0529
Iceland_PRS	BMI Raw	101304	1.825	0.0162	2761.4	0.1100
Iceland_GRS-with-Outliers	BMI Raw	101304	1.270	0.0166	1274.1	0.0540
Iceland_GRS	BMI Raw	101304	1.216	0.0166	1171.0	0.0498

Supplementary Table 1 All scores have been scaled to have variance 1. The BMI phenotype is described in the methods, essentially it is BMI corrected for standard covariates and inverse normal transformed. The raw BMI is the BMI at first visit in case of the UK Biobank data, for the Icelandic data, the raw BMI is the average of multiple measurments in case of many, the association is also adjusted for sex, yob and principal components. The statistical test used is a two-sided t-test. P-values are not adjusted for multiple comparisons.

Summary of BMI measurements

population	Mean BMI	SD BMI	BETA BMI phenotype regressed on raw BMI	BETA BMI inverse normal transformed regressed on raw BMI
Iceland	27.26	5.57	5.15	5.43
UKBiobank	27.42	4.77	4.56	4.64

Supplementary Table 2 The raw BMI and BMI phenotypes are described in methods. The BMI inverse normal transformed is the raw bmi inverse normal transformed. Thus the values in the last two columns show the difference in effect when we adjust for covariates or not.

Main mediation results with BMI-GRS score

Outcome	N cases	N controls	population	Score OR and 95% CI	-log10(p)	Score adj bmi OR and 95% CI	-log10(p adj)
Atrial fibrillation	11719	81147	Iceland	1.068, (1.049, 1.086)	13.0	1.0264, (1.0084, 1.045)	2.418
Chronic kidney disease	2753	88587	Iceland	1.089, (1.055, 1.125)	6.7	1.0519, (1.0178, 1.087)	2.584
Fatty liver disease	587	93828	Iceland	1.104, (1.033, 1.181)	2.4	0.9616, (0.8974, 1.030)	0.574
Glucose intolerance	478	90862	Iceland	1.174, (1.090, 1.265)	4.6	1.0736, (0.9947, 1.159)	1.167
Heart failure	11210	80130	Iceland	1.104, (1.084, 1.124)	24.9	1.0444, (1.0248, 1.064)	5.152
Hip replacement	2817	42406	Iceland	1.085, (1.050, 1.120)	6.1	1.0023, (0.9697, 1.036)	0.051
Knee replacement	3603	42406	Iceland	1.168, (1.135, 1.203)	25.0	1.0148, (0.9843, 1.046)	0.462
Myocardial Infarction	10609	82576	Iceland	1.060, (1.041, 1.079)	9.6	1.0272, (1.0084, 1.046)	2.364
Stroke	4615	88405	Iceland	1.047, (1.020, 1.074)	3.3	1.0352, (1.0086, 1.062)	2.041
Type 2 diabetes E11	11153	81662	Iceland	1.227, (1.207, 1.248)	127.9	1.0537, (1.0352, 1.072)	8.159
Atrial fibrillation	33733	395643	UKBiobank	1.081, (1.070, 1.092)	47.3	1.0168, (1.0058, 1.028)	2.595
Chronic kidney disease	15888	413812	UKBiobank	1.100, (1.084, 1.116)	36.2	1.0036, (0.9885, 1.019)	0.194
Fatty liver disease	7552	421824	UKBiobank	1.180, (1.156, 1.205)	54.2	0.9944, (0.9732, 1.016)	0.213
Glucose intolerance	11382	417994	UKBiobank	1.113, (1.094, 1.132)	34.0	0.9920, (0.9746, 1.010)	0.432
Heart failure	16096	413172	UKBiobank	1.165, (1.148, 1.182)	92.4	1.0637, (1.0477, 1.080)	14.884
Hip replacement	12585	417115	UKBiobank	1.087, (1.069, 1.104)	22.7	1.0283, (1.0112, 1.046)	2.951
Knee replacement	15564	414028	UKBiobank	1.176, (1.159, 1.194)	102.1	1.0106, (0.9951, 1.026)	0.740
Myocardial Infarction	25221	404047	UKBiobank	1.107, (1.094, 1.120)	62.9	1.0370, (1.0244, 1.050)	8.222
Stroke	3202	426066	UKBiobank	1.037, (1.004, 1.070)	1.6	1.0017, (0.9694, 1.035)	0.037
Type 2 diabetes E11	35663	393713	UKBiobank	1.263, (1.250, 1.276)	447.4	1.0392, (1.0280, 1.050)	11.483

Supplementary Table 3 Main mediation results for the BMI-GRS score with outliers excluded. We include grs score ORs and the grs score ORs adjusted for BMI. The odds ratios correspond to 1 unit increase in BMI. The statistical test used is a two-sided Wald test. P-values are not adjusted for multiple comparisons.

Mediation results stratified by sex in UK Biobank data with BMI-GRS score

Outcome	N cases	N controls	population	Score OR and 95% CI	-log10(p)	Score adj bmi OR and 95% CI	-log10(p adj)
Atrial fibrillation	12204	219633	UKBiobank females	1.077, (1.0602, 1.095)	19.15	1.0176, (1.0009, 1.034)	1.418
Chronic kidney disease	8403	223612	UKBiobank females	1.083, (1.0630, 1.104)	15.86	0.9910, (0.9718, 1.011)	0.433
Fatty liver disease	3816	228021	UKBiobank females	1.195, (1.1629, 1.229)	36.33	1.0048, (0.9765, 1.034)	0.129
Glucose intolerance	5336	226501	UKBiobank females	1.115, (1.0889, 1.141)	19.00	0.9958, (0.9720, 1.020)	0.133
Heart failure	5616	226165	UKBiobank females	1.167, (1.1406, 1.194)	38.78	1.0651, (1.0400, 1.091)	6.696
Hip replacement	7478	224537	UKBiobank females	1.068, (1.0471, 1.090)	10.00	1.0208, (1.0001, 1.042)	1.306
Knee replacement	8449	223510	UKBiobank females	1.167, (1.1451, 1.189)	56.88	0.9987, (0.9792, 1.019)	0.045
Myocardial Infarction	7243	224538	UKBiobank females	1.080, (1.0585, 1.102)	13.02	1.0130, (0.9920, 1.034)	0.645
Stroke	1225	230556	UKBiobank females	1.036, (0.9868, 1.087)	0.81	0.9933, (0.9451, 1.044)	0.102
Type 2 diabetes	13795	218042	UKBiobank females	1.264, (1.2457, 1.284)	206.01	1.0379, (1.0214, 1.055)	5.287
Atrial fibrillation	21520	175947	UKBiobank males	1.086, (1.0705, 1.102)	28.9	1.0168, (1.0018, 1.032)	1.56
Chronic kidney disease	7483	190130	UKBiobank males	1.121, (1.0959, 1.148)	21.7	1.0206, (0.9966, 1.045)	1.03
Fatty liver disease	3734	193733	UKBiobank males	1.165, (1.1282, 1.202)	20.2	0.9837, (0.9516, 1.017)	0.48
Glucose intolerance	6044	191423	UKBiobank males	1.113, (1.0855, 1.142)	16.1	0.9880, (0.9625, 1.014)	0.43
Heart failure	10476	186939	UKBiobank males	1.171, (1.1486, 1.195)	55.2	1.0662, (1.0447, 1.088)	9.15
Hip replacement	5107	192506	UKBiobank males	1.116, (1.0858, 1.147)	14.3	1.0402, (1.0111, 1.070)	2.19
Knee replacement	7114	190447	UKBiobank males	1.190, (1.1624, 1.219)	46.8	1.0284, (1.0035, 1.054)	1.60
Myocardial Infarction	17973	179442	UKBiobank males	1.127, (1.1095, 1.144)	51.8	1.0514, (1.0349, 1.068)	9.24
Stroke	1976	195439	UKBiobank males	1.040, (0.9952, 1.086)	1.1	1.0085, (0.9641, 1.055)	0.15
Type 2 diabetes	21860	175607	UKBiobank males	1.272, (1.2538, 1.290)	244.4	1.0421, (1.0265, 1.058)	7.06

Supplementary Table 4 Mediation results stratified by sex. We include grs score ORs and grs score ORs adjusted for BMI. The ORs correspond to 1 unit increase in BMI. The statistical test used is a two-sided Wald test. P-values are not adjusted for multiple comparisons.

Association of conservative BMI-GRS with raw BMI by sex

SEX	BETA GRS REGRESSED ON RAW BMI
MALES	1.01
FEMALES	1.17

Supplementary Table 5 Association of BMI-GRS with raw BMI by sex. These scaling parameters are used for the sex specific analysis.

Mediation analysis with GRS-with-Outliers, conservative GRS and outliers

Outcome	N cases	N controls	population	Score OR and 95% CI	-log10(p)	Score adj bmi OR and 95% CI	-log10(p adj)
Atrial fibrillation	11719	81147	Iceland	1.064, (1.047, 1.082)	12.7	1.0227, (1.0054, 1.040)	2.011
Chronic kidney disease	2753	88587	Iceland	1.076, (1.043, 1.110)	5.4	1.0383, (1.0059, 1.072)	1.695
Fatty liver disease	587	93828	Iceland	1.090, (1.022, 1.162)	2.0	0.9473, (0.8862, 1.013)	0.955
Glucose intolerance	478	90862	Iceland	1.169, (1.088, 1.255)	4.7	1.0677, (0.9920, 1.149)	1.093
Heart failure	11210	80130	Iceland	1.100, (1.081, 1.120)	25.2	1.0405, (1.0217, 1.060)	4.688
Hip replacement	2817	42406	Iceland	1.080, (1.047, 1.114)	6.0	0.9975, (0.9662, 1.030)	0.057
Knee replacement	3603	42406	Iceland	1.173, (1.141, 1.206)	28.3	1.0190, (0.9896, 1.049)	0.682
Myocardial Infarction	10609	82576	Iceland	1.047, (1.029, 1.065)	6.7	1.0138, (0.9960, 1.032)	0.887
Stroke	4615	88405	Iceland	1.038, (1.012, 1.063)	2.5	1.0258, (1.0004, 1.052)	1.333
Type 2 diabetes	11153	81662	Iceland	1.197, (1.178, 1.216)	107.4	1.0235, (1.0062, 1.041)	2.118
Atrial fibrillation	33733	395643	UKBiobank	1.078, (1.067, 1.089)	47.1	1.0137, (1.0032, 1.0244)	1.969
Chronic kidney disease	15888	413812	UKBiobank	1.091, (1.076, 1.107)	32.7	0.9949, (0.9804, 1.0096)	0.306
Fatty liver disease	7552	421824	UKBiobank	1.166, (1.143, 1.190)	50.4	0.9817, (0.9614, 1.0025)	1.075
Glucose intolerance	11382	417994	UKBiobank	1.096, (1.078, 1.114)	26.7	0.9750, (0.9584, 0.9918)	2.434
Heart failure	16096	413172	UKBiobank	1.158, (1.141, 1.174)	90.9	1.0564, (1.0410, 1.0720)	12.651
Hip replacement	12585	417115	UKBiobank	1.083, (1.067, 1.101)	22.7	1.0251, (1.0085, 1.0419)	2.548
Knee replacement	15564	414028	UKBiobank	1.176, (1.159, 1.193)	108.7	1.0094, (0.9944, 1.0246)	0.655
Myocardial Infarction	25221	404047	UKBiobank	1.089, (1.077, 1.102)	47.4	1.0187, (1.0066, 1.0308)	2.646
Stroke	3202	426066	UKBiobank	1.030, (0.999, 1.062)	1.2	0.9947, (0.9636, 1.0267)	0.131
Type 2 diabetes	35663	393713	UKBiobank	1.221, (1.209, 1.233)	352.5	0.9991, (0.9886, 1.0096)	0.066

Supplementary Table 6 Mediation results for the independent marker GRS score which includes outliers. We include grs score ORs and the grs score ORs adjusted for BMI. The odds ratios correspond to 1 unit increase in BMI. The statistical test used is a two-sided Wald test. P-values are not adjusted for multiple comparisons.

Mediation analysis with PRS, liberal score

Outcome	N cases	N controls	population	Score OR and 95% CI	-log10(p)	Score adj bmi OR and 95% CI	-log10(p adj)
Atrial fibrillation	11585	79266	Iceland	1.039, (1.0265, 1.051)	9.73	1.0030, (0.9910, 1.015)	0.2034
Chronic kidney disease	2727	86656	Iceland	1.057, (1.0345, 1.081)	6.27	1.0265, (1.0036, 1.050)	1.6399
Fatty liver disease	578	91857	Iceland	1.078, (1.0308, 1.128)	2.98	0.9556, (0.9113, 1.002)	1.2133
Glucose intolerance	472	88911	Iceland	1.117, (1.0630, 1.175)	4.88	1.0350, (0.9823, 1.091)	0.7068
Heart failure	11111	78272	Iceland	1.072, (1.0588, 1.086)	27.01	1.0226, (1.0094, 1.036)	3.1347
Hip replacement	2783	41557	Iceland	1.091, (1.0672, 1.115)	14.30	1.0196, (0.9968, 1.043)	1.0331
Knee replacement	2242	51028	Iceland	1.158, (1.1305, 1.186)	32.06	1.0458, (1.0197, 1.073)	3.2823
Myocardial Infarction	10496	80669	Iceland	1.035, (1.0226, 1.048)	7.59	1.0076, (0.9950, 1.020)	0.6244
Stroke	4555	86446	Iceland	1.011, (0.9934, 1.028)	0.65	1.0001, (0.9824, 1.018)	0.0023
Type 2 diabetes E11	10985	79815	Iceland	1.157, (1.1437, 1.170)	141.44	1.0099, (0.9977, 1.022)	0.9513
Atrial fibrillation	33733	395643	UKBiobank	1.085, (1.076, 1.094)	83.7	1.0198, (1.0110, 1.0287)	5.03
Chronic kidney disease	15888	413812	UKBiobank	1.108, (1.095, 1.121)	66.7	1.0079, (0.9956, 1.0202)	0.68
Fatty liver disease	7552	421824	UKBiobank	1.163, (1.144, 1.182)	71.8	0.9721, (0.9553, 0.9892)	2.84
Glucose intolerance	11382	417994	UKBiobank	1.082, (1.067, 1.096)	29.6	0.9563, (0.9428, 0.9700)	9.11
Heart failure	16096	413172	UKBiobank	1.140, (1.126, 1.153)	108.3	1.0364, (1.0238, 1.0491)	8.04
Hip replacement	12585	417115	UKBiobank	1.076, (1.063, 1.090)	28.4	1.0163, (1.0026, 1.0301)	1.71
Knee replacement	15564	414028	UKBiobank	1.190, (1.176, 1.204)	186.7	1.0171, (1.0045, 1.0298)	2.12
Myocardial Infarction	25221	404047	UKBiobank	1.077, (1.067, 1.088)	54.3	1.0051, (0.9953, 1.0151)	0.51
Stroke	3202	426066	UKBiobank	1.052, (1.025, 1.078)	4.1	1.0160, (0.9896, 1.0431)	0.62
Type 2 diabetes E11	35663	393713	UKBiobank	1.225, (1.215, 1.234)	538.9	0.9940, (0.9854, 1.0027)	0.75

Supplementary Table 7 Mediation results for the 611K marker PRS score, the most liberal score of the ones we tried. We include grs score ORs and the grs score ORs adjusted for BMI. The odds ratios correspond to 1 unit increase in BMI. The statistical test used is a two-sided Wald test. P-values are not adjusted for multiple comparisons.

Proportion Mediation by Scores

Outcome	population	GRS<br>Conservative score<br>proportion mediated	GRS-with-Outliers<br>Conservative score + outliers<br>proportion mediated	PRS<br>Liberal score<br>proportion mediated
Atrial fibrillation	Iceland	0.72 (0.57, 0.96)	0.77 (0.61, 1.03)	1.02 (0.82, 1.33)
Chronic kidney disease	Iceland	0.49 (0.33, 0.67)	0.57 (0.38, 0.79)	0.41 (0.31, 0.52)
Fatty liver disease	Iceland	1.40 (0.80, 5.69)	1.63 (0.89, 7.26)	1.13 (0.79, 1.91)
Glucose intolerance	Iceland	0.59 (0.37, 1.30)	0.62 (0.39, 1.37)	0.81 (0.51, 1.60)
Heart failure	Iceland	0.63 (0.54, 0.79)	0.66 (0.57, 0.82)	0.57 (0.51, 0.65)
Hip replacement	Iceland	1.03 (0.74, 1.63)	1.13 (0.79, 1.84)	1.00 (0.77, 1.32)
Knee replacement	Iceland	0.94 (0.81, 1.17)	0.93 (0.80, 1.14)	0.84 (0.76, 0.96)
Myocardial Infarction	Iceland	0.60 (0.46, 0.82)	0.76 (0.57, 1.13)	0.44 (0.37, 0.54)
Stroke	Iceland	0.29 (0.17, 0.79)	0.34 (0.20, 1.18)	0.43 (0.25, 1.21)
Type 2 diabetes E11	Iceland	0.76 (0.70, 0.82)	0.87 (0.80, 0.95)	0.86 (0.80, 0.91)
Atrial fibrillation	UKBiobank	0.79 (0.70, 0.91)	0.82 (0.72, 0.94)	0.74 (0.67, 0.82)
Chronic kidney disease	UKBiobank	0.96 (0.85, 1.15)	1.06 (0.92, 1.27)	0.92 (0.83, 1.05)
Fatty liver disease	UKBiobank	1.04 (0.92, 1.19)	1.13 (1.00, 1.30)	1.19 (1.06, 1.33)
Glucose intolerance	UKBiobank	1.08 (0.92, 1.28)	1.28 (1.07, 1.57)	1.48 (1.26, 1.78)
Heart failure	UKBiobank	0.61 (0.56, 0.68)	0.63 (0.58, 0.71)	0.71 (0.66, 0.79)
Hip replacement	UKBiobank	0.66 (0.55, 0.84)	0.69 (0.57, 0.87)	0.75 (0.63, 0.92)
Knee replacement	UKBiobank	0.94 (0.87, 1.03)	0.95 (0.87, 1.04)	0.90 (0.84, 0.96)
Myocardial Infarction	UKBiobank	0.65 (0.58, 0.74)	0.79 (0.70, 0.92)	0.88 (0.79, 1.00)
Stroke	UKBiobank	0.96 (0.29, 4.95)	1.15 (-2.78, 11.73)	0.79 (0.45, 1.95)
Type 2 diabetes E11	UKBiobank	0.85 (0.82, 0.89)	1.01 (0.96, 1.06)	1.02 (0.99, 1.07)

Supplementary Table 8 Mediation results for all three scores, the proportion mediated by BMI measurements, proportion and 95% CIs.

Association of BMI phenotype with outcomes

Outcome	population	N cases	N controls	OR and 95% CI	-log10(p)
Atrial fibrillation	UKB	33733	395643	1.068, (1.065, 1.071)	538.1
Chronic kidney disease	UKB	15888	413812	1.104, (1.100, 1.108)	600.6
Fatty liver disease	UKB	7552	421824	1.186, (1.180, 1.192)	921.7
Glucose intolerance	UKB	11382	417994	1.126, (1.121, 1.131)	655.7
Heart failure	UKB	16096	413172	1.106, (1.102, 1.110)	631.3
Hip replacement	UKB	12585	417115	1.063, (1.058, 1.067)	191.3
Knee replacement	UKB	15564	414028	1.179, (1.174, 1.184)	1546.3
Myocardial Infarction	UKB	25221	404047	1.073, (1.070, 1.076)	485.9
Stroke	UKB	3202	426066	1.037, (1.029, 1.046)	19.5
Type 2 diabetes E11	UKB	35663	393713	1.247, (1.243, 1.250)	5034.2
Atrial fibrillation	Iceland	11719	81147	1.057, (1.051, 1.062)	108.7
Chronic kidney disease	Iceland	2753	88587	1.055, (1.045, 1.065)	28.8
Fatty liver disease	Iceland	587	93828	1.147, (1.129, 1.166)	61.6
Glucose intolerance	Iceland	478	90862	1.117, (1.096, 1.140)	27.6
Heart failure	Iceland	11210	80130	1.086, (1.080, 1.091)	198.2
Hip replacement	Iceland	2817	42406	1.110, (1.100, 1.120)	115.2
Knee replacement	Iceland	3603	42406	1.201, (1.191, 1.211)	392.5
Myocardial Infarction	Iceland	10609	82576	1.045, (1.040, 1.051)	64.2
Stroke	Iceland	4615	88405	1.018, (1.011, 1.025)	5.9
Type 2 diabetes E11	Iceland	11153	81662	1.212, (1.206, 1.217)	1301.2

Supplementary Table 9 Associations of BMI phenotype with outcomes in Icelandic and UK data. The statistical test used is a two-sided Wald test. P-values are not adjusted for multiple comparisons.

Association of BMI phenotype with outcomes stratified by BMI at first visit to UK Biobank

Outcome	Strata	OR	LOWER 95% CI	UPPER 95% CI
Atrial fibrillation	All data	1.068	1.071	1.065
Chronic kidney disease	All data	1.104	1.108	1.100
Fatty liver disease	All data	1.186	1.192	1.180
Glucose intolerance	All data	1.126	1.131	1.121
Heart failure	All data	1.106	1.110	1.102
Hip replacement	All data	1.063	1.067	1.058
Knee replacement	All data	1.179	1.184	1.174
Myocardial Infarction	All data	1.073	1.076	1.070
Stroke	All data	1.038	1.046	1.030
Type 2 diabetes E11	All data	1.247	1.250	1.243
Atrial fibrillation	BMI > 25	1.104	1.108	1.099
Chronic kidney disease	BMI > 25	1.117	1.123	1.112
Fatty liver disease	BMI > 25	1.172	1.180	1.165
Glucose intolerance	BMI > 25	1.129	1.136	1.123
Heart failure	BMI > 25	1.153	1.159	1.147
Hip replacement	BMI > 25	1.058	1.065	1.052
Knee replacement	BMI > 25	1.167	1.173	1.162
Myocardial Infarction	BMI > 25	1.083	1.088	1.078
Stroke	BMI > 25	1.055	1.068	1.043
Type 2 diabetes E11	BMI > 25	1.261	1.266	1.257
Atrial fibrillation	25 < BMI < 35	1.081	1.087	1.075
Chronic kidney disease	25 < BMI < 35	1.114	1.123	1.105
Fatty liver disease	25 < BMI < 35	1.207	1.221	1.194
Glucose intolerance	25 < BMI < 35	1.142	1.152	1.132
Heart failure	25 < BMI < 35	1.122	1.131	1.113
Hip replacement	25 < BMI < 35	1.064	1.074	1.054
Knee replacement	25 < BMI < 35	1.185	1.195	1.176
Myocardial Infarction	25 < BMI < 35	1.088	1.095	1.081
Stroke	25 < BMI < 35	1.054	1.073	1.036
Type 2 diabetes E11	25 < BMI < 35	1.260	1.268	1.253

Supplementary Table 10 BMI phenotype associations in UK data, stratified by BMI at first visi

Supplementary References

1. Thorolfsdottir, R.B. *et al.* Coding variants in RPL3L and MYZAP increase risk of atrial fibrillation. *Communications biology* **1**, 68 (2018).
2. Sveinbjornsson, G. *et al.* Multiomics study of nonalcoholic fatty liver disease. *Nature Genetics*, 1-12 (2022).
3. Styrkarsdottir, U. *et al.* The CRTAC1 Protein in Plasma Is Associated With Osteoarthritis and Predicts Progression to Joint Replacement: A Large-Scale Proteomics Scan in Iceland. *Arthritis & Rheumatology* **73**, 2025-2034 (2021).
4. Helgadottir, A. *et al.* Cholesterol not particle concentration mediates the atherogenic risk conferred by apolipoprotein B particles: a Mendelian randomization analysis. *European Journal of Preventive Cardiology* **29**, 2374-2385 (2022).
5. Malik, R. *et al.* Multiancestry genome-wide association study of 520,000 subjects identifies 32 loci associated with stroke and stroke subtypes. *Nature genetics* **50**, 524-537 (2018).
6. Jonsdottir, G.A. *et al.* Genetic propensities for verbal and spatial ability have opposite effects on body mass index and risk of schizophrenia. *Intelligence* **88**, 101565 (2021).
7. Yengo, L. *et al.* Meta-analysis of genome-wide association studies for height and body mass index in∼ 700000 individuals of European ancestry. *Human molecular genetics* **27**, 3641-3649 (2018).
8. Verbanck, M., Chen, C.-Y., Neale, B. & Do, R. Detection of widespread horizontal pleiotropy in causal relationships inferred from Mendelian randomization between complex traits and diseases. *Nature genetics* **50**, 693-698 (2018).
