# Supplementary material for: Sequence variants associated with BMI affect disease risk through BMI itself
Source: Nat Commun. 2024 Nov 12;15:9335. doi: 10.1038/s41467-024-53568-9 (PMC11557886; doi:10.1038/s41467-024-53568-9)
Supplement: Supplementary file 3 — Description of additional supplementary files [file 41467_2024_53568_MOESM3_ESM.pdf]

# Description of Additional Supplementary files

**Supplementary Data 1 -** SNPs used to create the GRS score along with annotations on which were classified as outliers in the score disease associations.
