# Supplementary material for: Sequence variants associated with BMI affect disease risk through BMI itself
Source: Nat Commun. 2024 Nov 12;15:9335. doi: 10.1038/s41467-024-53568-9 (PMC11557886; doi:10.1038/s41467-024-53568-9)
Supplement: Supplementary file 5 — Reporting Summary [file 41467_2024_53568_MOESM5_ESM.pdf]

nature portfolio

Corresponding author(s): Kari Stefansson

Last updated by author(s): Jul 4, 2024

Reporting Summary

Nature Portfolio wishes to improve the reproducibility of the work that we publish. This form provides structure for consistency and transparency in reporting. For further information on Nature Portfolio policies, see our Editorial Policies and the Editorial Policy Checklist.

Statistics

For all statistical analyses, confirm that the following items are present in the figure legend, table legend, main text, or Methods section.

n/a	Confirmed	
☐	☒	The exact sample size (*n*) for each experimental group/condition, given as a discrete number and unit of measurement
☐	☒	A statement on whether measurements were taken from distinct samples or whether the same sample was measured repeatedly
☐	☒	The statistical test(s) used AND whether they are one- or two-sided<br>*Only common tests should be described solely by name; describe more complex techniques in the Methods section.*
☐	☒	A description of all covariates tested
☐	☒	A description of any assumptions or corrections, such as tests of normality and adjustment for multiple comparisons
☐	☒	A full description of the statistical parameters including central tendency (e.g. means) or other basic estimates (e.g. regression coefficient) AND variation (e.g. standard deviation) or associated estimates of uncertainty (e.g. confidence intervals)
☐	☒	For null hypothesis testing, the test statistic (e.g. *F*, *t*, *r*) with confidence intervals, effect sizes, degrees of freedom and *P* value noted<br>*Give P values as exact values whenever suitable.*
☒	☐	For Bayesian analysis, information on the choice of priors and Markov chain Monte Carlo settings
☒	☐	For hierarchical and complex designs, identification of the appropriate level for tests and full reporting of outcomes
☒	☐	Estimates of effect sizes (e.g. Cohen's *d*, Pearson's *r*), indicating how they were calculated

Our web collection on statistics for biologists contains articles on many of the points above.

Software and code

Policy information about availability of computer code

Data collection: *No software was used for data collection.*

Data analysis:
We used R, version 3.6.3 for analysis and visualizations
R package mediation (v4.5.0)
R package tidyverse (v1.3.0)
R package ggsci (v2.9)
R package ggrepel (v0.8.2)
Ensembl version 87, https://www.ensembl.org/index.html
Graphtyper version 2, https://github.com/DecodeGenetics/graphtyper;
BOLT-LMM version 2.1, https://data.broadinstitute.org/alkesgroup/BOLT-LMM/downloads/;
IMPUTE2 version 2.3.1, https://mathgen.stats.ox.ac.uk/impute/impute_v2.html;
dbSNP version 140, http://www.ncbi.nlm.nih.gov/SNP/;

For manuscripts utilizing custom algorithms or software that are central to the research but not yet described in published literature, software must be made available to editors and reviewers. We strongly encourage code deposition in a community repository (e.g. GitHub). See the Nature Portfolio guidelines for submitting code & software for further information.

Data

Policy information about availability of data

All manuscripts must include a data availability statement. This statement should provide the following information, where applicable:

- Accession codes, unique identifiers, or web links for publicly available datasets
- A description of any restrictions on data availability
- For clinical datasets or third party data, please ensure that the statement adheres to our policy

The SNPs used to construct the BMI-GRS scores can be found in the Supplementary Data 1 file along with information on which are classified as outliers. The Icelandic individual level data cannot be made publicly available due to data privacy laws. The UK Biobank data were downloaded under application no. 56270. Individual-level genomic and phenotypic data from the UK Biobank are available to researchers upon application (https://ukbiobank.ac.uk). Source data are provided as a Source Data file.

Research involving human participants, their data, or biological material

Policy information about studies with human participants or human data. See also policy information about sex, gender (identity/presentation), and sexual orientation and race, ethnicity and racism.

Reporting on sex and gender	Adjustments are made with biological sex as a covariate and we also perform stratified analysis on biological sex. We always use the word sex when reporting these results.
Reporting on race, ethnicity, or other socially relevant groupings	We restricted our analysis to individuals of European descent.
Population characteristics	UK Biobank:<br>The UK Biobank (UKB) is a vast biomedical repository holding intricate phenotypic and genetic data on about 500,000 individuals from England, Wales, and Scotland. We restricted our analysis to individuals of European descent. They were between 40-69 years old at the point of entry. 46% of the recruited were male and 54% female. 57% were aged 40-59 years and 43% aged 60-69.<br><br>Icelandic population:<br>The Icelandic deCODE database contains a collection of data from Icelanders that have participated in several genetic initiatives. Whole-genome sequencing data is available for 49,708 Icelander where variants identified through whole-genome sequencing were imputed into 155,250 chip-genotypes Icelanders as well as their close relatives based on genealogy.
Recruitment	For the deCODE Genetics study, individuals were recruited through various research projects at deCODE Genetics, encompassing a significant portion of the adult Icelandic population. The UK Biobank contains data on half a million participants from across the UK, all of whom were recruited through specially designed assessment centers.
Ethics oversight	All participants that donated biological samples signed informed consent for research. To ensure participant confidentiality, a sanctioned encryption system was employed, monitored by the Icelandic Data Protection Authority. The study was approved by the Icelandic National Bioethics Committee (approval No. VSN-17-076).<br><br>For the UK data all participants willingly gave their informed consent. The operational methodologies were vetted and sanctioned by The North West Research Ethics Committee, and access to the UKB resources was gained through application no. #56270.

Note that full information on the approval of the study protocol must also be provided in the manuscript.

Field-specific reporting

Please select the one below that is the best fit for your research. If you are not sure, read the appropriate sections before making your selection.

☒ Life sciences ☐ Behavioural & social sciences ☐ Ecological, evolutionary & environmental sciences

For a reference copy of the document with all sections, see nature.com/documents/nr-reporting-summary-flat.pdf

Life sciences study design

All studies must disclose on these points even when the disclosure is negative.

Sample size	Sample sizes are reported in the article for each specific analyses and were based on the available data in each population.
Data exclusions	The data was restricted to data from individuals of European ancestry.
Replication	*The results are computed in Icelandic and UK data. Results are largely consistent and inconsistancies are discussed.*

Randomization | Not applicatble (GWAS study, not a randomized trial)

Blinding | Not applicable (GWAS study, not a randomized trial, so no blinding is required)

Reporting for specific materials, systems and methods

We require information from authors about some types of materials, experimental systems and methods used in many studies. Here, indicate whether each material, system or method listed is relevant to your study. If you are not sure if a list item applies to your research, read the appropriate section before selecting a response.

Materials & experimental systems

n/a	Involved in the study
☒	☐ Antibodies
☒	☐ Eukaryotic cell lines
☒	☐ Palaeontology and archaeology
☒	☐ Animals and other organisms
☒	☐ Clinical data
☒	☐ Dual use research of concern
☒	☐ Plants

Methods

n/a	Involved in the study
☒	☐ ChIP-seq
☒	☐ Flow cytometry
☒	☐ MRI-based neuroimaging

Plants

Seed stocks | *Report on the source of all seed stocks or other plant material used. If applicable, state the seed stock centre and catalogue number. If plant specimens were collected from the field, describe the collection location, date and sampling procedures.*

Novel plant genotypes | *Describe the methods by which all novel plant genotypes were produced. This includes those generated by transgenic approaches, gene editing, chemical/radiation-based mutagenesis and hybridization. For transgenic lines, describe the transformation method, the number of independent lines analyzed and the generation upon which experiments were performed. For gene-edited lines, describe the editor used, the endogenous sequence targeted for editing, the targeting guide RNA sequence (if applicable) and how the editor was applied.*

Authentication | *Describe any authentication procedures for each seed stock used or novel genotype generated. Describe any experiments used to assess the effect of a mutation and, where applicable, how potential secondary effects (e.g. second site T-DNA insertions, mosiacism, off-target gene editing) were examined.*
